# Recent declines in salmon body size impact ecosystems and fisheries

**DOI:** 10.1038/s41467-020-17726-z

**Published:** 2020-08-19

**Authors:** K. B. Oke, C. J. Cunningham, P. A. H. Westley, M. L. Baskett, S. M. Carlson, J. Clark, A. P. Hendry, V. A. Karatayev, N. W. Kendall, J. Kibele, H. K. Kindsvater, K. M. Kobayashi, B. Lewis, S. Munch, J. D. Reynolds, G. K. Vick, E. P. Palkovacs

**Affiliations:** 1 Department of Ecology and Evolutionary Biology, University of California, Santa Cruz, CA 95060 USA; 2 College of Fisheries and Ocean Sciences, University of Alaska Fairbanks, Juneau, AK 99801 USA; 3 Fisheries, Aquatic Science & Technology Laboratory, Alaska Pacific University, Anchorage, AK 99508 USA; 4 College of Fisheries and Ocean Sciences, University of Alaska Fairbanks, Fairbanks, AK 99775 USA; 5 Department of Environmental Science and Policy, University of California, Davis, CA 95616 USA; 6 Environmental Science, Policy, and Management, University of California, Berkeley, CA 94720 USA; 7 National Center for Ecological Analysis and Synthesis, University of California, Santa Barbara, CA 93101 USA; 8 Department of Biology and Redpath Museum, McGill University, Montreal, QC H3A 2K6 Canada; 9 Washington Department of Fish and Wildlife, Olympia, WA 98501 USA; 10 Department of Fish and Wildlife Conservation, Virginia Polytechnic Institute and State University, Blacksburg, VA 24061 USA; 11 Division of Commercial Fisheries, Alaska Department of Fish and Game, Anchorage, AK 99518 USA; 12 National Marine Fisheries Service, Fisheries Ecology Division, Southwest Fisheries Science Center, Santa Cruz, CA 95060 USA; 13 Earth to Ocean Research Group, Department of Biological Sciences, Simon Fraser University, Burnaby, BC V5A 1S6 Canada; 14 GKV & Sons, Contracting to Tanana Chiefs Conference, Fairbanks, AK 99709 USA

**Keywords:** Climate-change ecology, Evolutionary ecology, Conservation biology, Ecosystem services

## Abstract

Declines in animal body sizes are widely reported and likely impact ecological interactions and ecosystem services. For harvested species subject to multiple stressors, limited understanding of the causes and consequences of size declines impedes prediction, prevention, and mitigation. We highlight widespread declines in Pacific salmon size based on 60 years of measurements from 12.5 million fish across Alaska, the last largely pristine North American salmon-producing region. Declines in salmon size, primarily resulting from shifting age structure, are associated with climate and competition at sea. Compared to salmon maturing before 1990, the reduced size of adult salmon after 2010 has potentially resulted in substantial losses to ecosystems and people; for Chinook salmon we estimated average per-fish reductions in egg production (−16%), nutrient transport (−28%), fisheries value (−21%), and meals for rural people (−26%). Downsizing of organisms is a global concern, and current trends may pose substantial risks for nature and people.

## Abstract (web summary)

The average body size of salmon has declined rapidly over recent decades. Here the authors quantify changes in body size distributions for Pacific salmon in Alaska and examine the causes and consequences of size declines for ecosystems, food security, and commercial fisheries.

## Introduction

Few organismal traits are as profoundly important as body size, given its role in reproductive fitness, physiology, demography, predator–prey dynamics, and value for human use^1^. Yet major selective forces such as climate change and harvest may be causing widespread declines in organismal body size^2–5^. Climate change has been linked to body size declines in many species^2,3^, including Soay sheep in Scotland^6^, aquatic ectotherms across Europe^7^, and migratory North American birds^8^. Harvest is also known to result in smaller body size^5,9^, for example, declines in body size and age-at-maturity preceded the collapse of Atlantic cod stocks off the eastern coast of Canada^10^. Understanding the causes of body size declines is daunting given the influence of numerous, potentially interacting factors. Individually or in unison, these underlying factors can influence body size through shifting population age structure, changing growth rates, or a combination thereof. Age truncation can compound the effects of body size on population productivity by increasing demographic variability in response to changing environments^11^. Body size declines influence species’ demography^4^ and trophic interactions^12^ and may reduce the sustainable delivery of ecosystem services such as fisheries yield^9^.

Here, we examine changes in body size for four species of Pacific salmon (*Oncorhynchus* spp.), by assembling a 60-year (1957–2018) database of size and age measurements from 12.5 million individually-measured fish. The uniquely large spatial and temporal scale of our dataset enabled us to conduct one of the most comprehensive studies to quantify system-wide body size declines across multiple species and identify potential causal mechanisms, and one of the first studies to quantify ecological and socioeconomic consequences of those observed size declines. Our overarching goals were to understand the magnitude and consistency of size declines across regions and species, evaluate potential causes, and quantify the consequences of these changes for ecosystems and people.

Pacific salmon are integral ecosystem components and contribute to human well-being, primarily as sources of food security and cultural connection^13,14^. The annual return of salmon to their natal streams provides vital nutrient subsidies that support freshwater, riparian, and terrestrial ecosystems^15^. Alaska is widely considered a stronghold of intact, functioning salmon–people ecosystems, largely free of the factors that have severely depressed salmon abundances elsewhere, such as over-harvest, habitat-loss, net pen aquaculture (prohibited by law in Alaska), dams, and water diversion^16^. However, accumulating evidence from local and indigenous knowledge suggests that adult salmon body sizes are decreasing, including in Alaska where salmon provide critical support for ecosystems and people^17–19^, cf. ref. ^20^.

Serious consequences for ecosystems and people could result from salmon size declines. Smaller salmon transport less marine-derived nutrients and produce fewer offspring^21,22^. Smaller salmon could threaten food security in rural salmon-dependent communities, where diminished access to calorie-rich salmon directly influences well-being and human health^13^. From an economic perspective, smaller salmon translate to lost commercial fisheries profit due to reduced flesh recovery rates (proportionally more skin, viscera, and bones but less muscle), increased processing cost, and lower prices. In some cases, losses due to changing salmon size could be mitigated by increasing conspecific abundances for certain ecosystems services and species. However, the opportunity for mitigation will be limited for species like Chinook salmon that have generally experienced declines in abundance concurrent with size declines^23^ or for ecosystem services for which abundance cannot replace size. For example, recreational anglers highly value catching large fish, which influences decisions on fishing trip destinations^24^. In addition, abundant species like sockeye and pink salmon cannot replace many ecosystem services provided by Chinook salmon because Chinook salmon generally have much greater migration distances, fat content, and cultural importance. For salmon in Alaska, the extent to which body size is changing across species and regions, the causes of size changes, and the consequences for nature and people are poorly known.

We synthesize patterns of salmon body size change across the state of Alaska for Chinook (*Oncorhynchus tshawytscha*), chum (*O. keta*), coho (*O. kisutch*), and sockeye salmon (*O. nerka*). While previous studies have documented changes in size and age in Pacific salmon^17,18,20^, our investigation across species, decades, and locations allows a uniquely comprehensive analysis of consistency in trends, causes, and consequences of those changes at an unprecedented spatial and temporal scale. Our analysis is based on six decades of salmon size and age measurements collected by the Alaska Department of Fish and Game from 1014 sampling locations across Alaska’s diverse landscapes—from temperate rainforests to Arctic ecosystems.

We show that body size has declined significantly across Pacific salmon species in Alaska, but that the rate of change has not been constant over time. Changing age structure (younger age-at-maturity) consistently explains a greater proportion of overall size changes than do changing growth rates (smaller size-at-age); salmon are getting smaller primarily because they are returning to reproduce at a younger age than they did in the past. Climate change and competition with highly abundant wild and hatchery-produced salmon appear to be widespread drivers of size declines. We found  limited evidence for a widespread role of size-selective harvest. The consequences of these changes for ecosystems and people are widespread: size declines are likely causing decreases in key ecological processes and human uses, including per-capita egg production, marine-derived nutrient subsidies, rural food security, and commercial value for harvesters.

## Results

### Consistency in salmon size declines

**Fig. 1:**
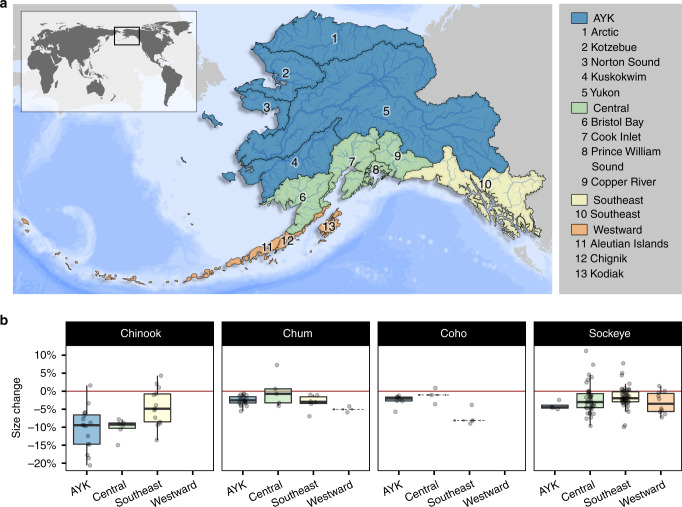
Across Alaska, average salmon body size has gotten smaller. On average, salmon body size was smaller post-2010 compared with pre-1990 across all areas and species examined. **a** Map of sampling area with regions numbered and colored by Alaska Department of Fish and Game management area. Our analyses included data from all regions shown except Arctic. **b** Boxplots show percent change in mean length between data collected before 1990 and after 2010. Points show change in mean length for individual populations. Red line indicates no change. Center line represents the median, box limits represent the upper and lower quantiles, whiskers represent the 1.5× interquartile range. Only populations for which we had data in both periods were included (100 sockeye, 34 Chinook, 32 chum, and 13 coho salmon populations). If sufficient data were available for three or fewer populations, the box was replaced by a gray dashed line at the median. AYK represents the Arctic–Yukon–Kuskokwim management area. Sample sizes are presented in Supplementary Data 4.

In all four salmon species, average body sizes were smaller after 2010 compared to before 1990 (the earliest baseline with sufficient data, Fig. 1). Comparing mean body length pre-1990 to mean body length post-2010, Chinook salmon exhibited the greatest magnitude decline, averaging an 8.0% decline in body length, compared to 3.3% in coho salmon, 2.4% in chum salmon, and 2.1% in sockeye salmon. Within species, the magnitude of declines varied among regions and populations (Fig. 1). For example, Chinook salmon populations in Westward Alaska and Arctic–Yukon–Kuskokwim declined by 10% on average, whereas conspecifics in Southeast Alaska declined by 4%.

**Fig. 2:**
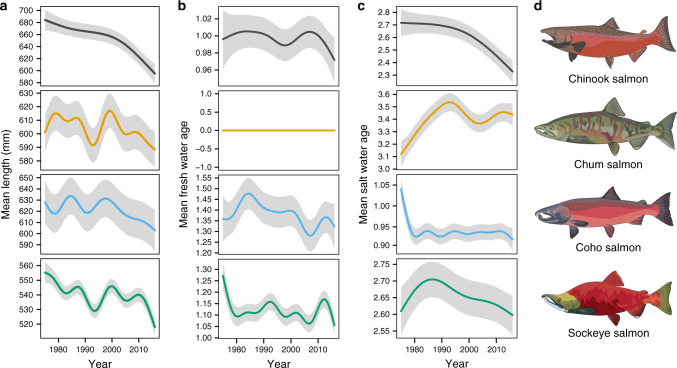
Body size declines are significant and nonlinear. **a** Mean fish length has changed in a nonlinear pattern, as demonstrated by the nonlinear year effect from GAMs on mean population length with fixed effects of region and population. **b** Mean freshwater age (in years) has generally declined, except for chum salmon, which leave freshwater shortly after emergence. **c** Mean saltwater age (in years) has also generally declined, except in chum salmon, which increased in saltwater age until around 1990, then decreased. Plots are conditioned on reference populations with the longest time series for each species, but the pattern plotted is the common pattern through time calculated for all populations. Gray areas represent 95% confidence intervals for the nonlinear year effect. **d** Male salmon in spawning coloration. Sample sizes are presented in Supplementary Data 5.

General additive models (GAMs) confirmed that average sizes declined through time in each species (nonlinear year effect for each species *p* < 0.0001, *R*^2^ = 0.453, 0.621, 0.687, 0.784 for Chinook, sockeye, coho, and chum salmon respectively, Fig. 2a), although the common (among location) pattern in average size across time differed between species. To evaluate whether there was greater support for species-specific nonlinear year effects through time, or a single shared temporal pattern, we fit competing GAMs to mean-variance standardized length observations from each location. Inclusion of species-specific nonlinear year effects explained much more variance (*R*^2^ = 0.80) compared to a single shared (i.e., shared among species) nonlinear year effect (*R*^2^ = 0.04). This result was confirmed by fitting an additional model that included both the common and species-specific nonlinear year effects, in which species-specific trends were significant (*p* < 0.0001) while the common trend was not (*p* = 0.3). All species are declining in body size but patterns of decline differ among species, thus species-specific trends were analyzed and are discussed separately.

Within each species, size trends were nonlinear (effective degrees of freedom = 3.75 for Chinook, 8.86 for chum, 7.78 for coho, and 8.81 for sockeye salmon; Fig. 2a) and included several periods of increasing and decreasing size. Separate species-specific models (Fig. 2a) revealed similarities among sockeye, chum, and coho salmon, including shared size declines starting in the mid-1980s followed by recovery in the early-1990s. These three species all showed an abrupt decline in body size starting in 2000 and intensifying after 2010. Size declines were more linear in Chinook salmon than in other species, but the rate of decline also accelerated after 2000.

Comparing model fits for GAMs that incorporate regional- and population-level trends revealed that Chinook and coho salmon exhibit high spatial variation in patterns of body size change, best explained by population-specific nonlinear year effects. In contrast, sockeye and chum salmon populations exhibited less spatial variability, which was best explained by regional-level patterns (Supplementary Table S1).

### Contributions of declining age versus growth

**Fig. 3:**
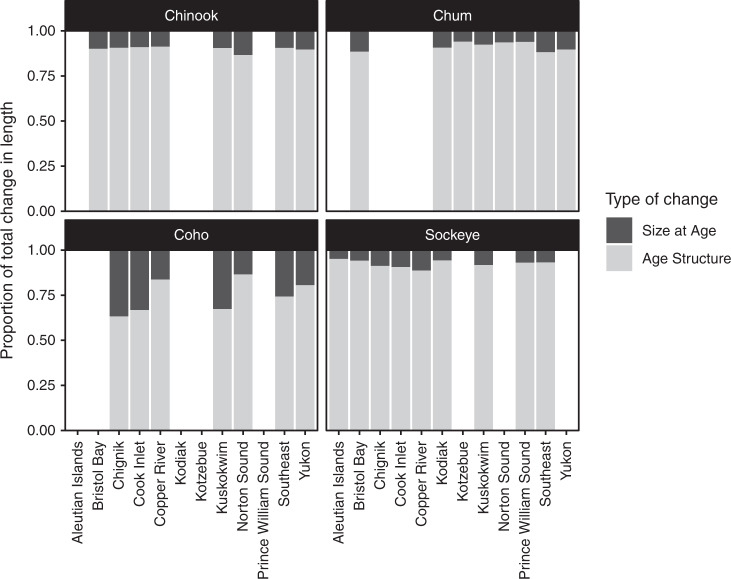
Body size declines result primarily from shifting age structure. Changes in population mean length are primarily due to changing age composition (gray) and to a much lesser extent changing size-at-age (black). For each population the mean among-year contribution was calculated, then region means calculated from population-level means. Sample sizes are presented in Supplementary Data 6.

Across species, shifts in age structure explained 88% of interannual variation in mean size on average (Fig. 3). In general, salmon are currently smaller than in the past because adults are returning to spawn at younger ages (Fig. 2). Changing size-at-age (Supplementary Fig. S1), which might result from decreased growth, explained a greater proportion of size change in coho salmon (20% on average) than in other species (7.4% in Chinook salmon, 7.1% in chum salmon, 5.9% in sockeye salmon), yet across all species and regions the contribution of changing size-at-age to declines in body sizes was less important than that of changing age structure.

### Causes of salmon size declines

Both environmental change and increased competition at sea with highly abundant wild and hatchery salmon could result in body size declines through reductions in the availability or quality of food resources^18,20^. Climate warming might also reduce ectotherm body size by increasing metabolic and developmental rates^2^. Finally, all of these environmental factors could result in increased natural mortality in the ocean, leading to reduced average age-at-return to freshwater.

To evaluate the hypothesized effects of climate and competition at sea (Supplementary Figs. S2, S3), we fit hierarchical Bayesian models estimating the association between temporal trends in location-specific salmon size and a range of environmental covariates, while also estimating a nonlinear year effect describing temporal trends in length that were common across populations but not explained by covariates. After accounting for absolute body size differences among populations, our ability to explain changes in body size ranged from a Bayesian^25^ *R*^2^ of 0.28 in sockeye salmon, 0.29 in Chinook salmon, 0.35 in chum salmon, to 0.48 in coho salmon.

**Fig. 4:**
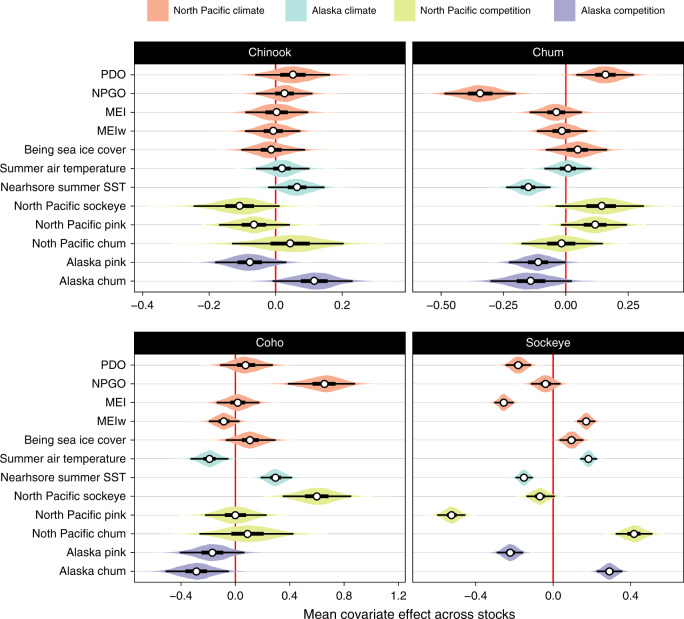
Climate and competition influence salmon body size. Effects of climate and competition proxies (detailed in Methods, MEIw is winter MEI) on body size varied among species, as estimated by hierarchical Bayesian models describing length-environment relationships. Posterior probability distributions (in color) for estimated species-specific (group) mean effects of climate and competition covariates across locations. Posterior medians, 50% and 95% credible intervals are described by the white point, thick and thin black lines. Negative effects indicate high values of a covariate are correlated with smaller salmon body size on average across locations in Alaska. See Supplementary Fig. S4 for population-specific covariate effect estimates. Sample sizes are presented in Supplementary Data 7.

Multiple factors with small individual effects were associated with body size declines (Fig. 4). Although the relative importance of each metric differed among species (Fig. 4) and populations (Supplementary Fig. S4), at least one climate metric and one competition metric were important for each species. Only Alaskan pink salmon abundance had a negative association with body size across all species, but the negative association was weak in all cases except sockeye salmon. Some factors emerged as particularly important for individual species. For sockeye salmon, North Pacific pink salmon abundance had a particularly strong negative association with body size. For chum salmon, a strong negative association with the North Pacific Gyre Oscillation (NPGO) contrasted with a similarly strong positive association for coho salmon. No single factor was a particularly important predictor of body size in Chinook salmon; instead many factors had moderate contributions to body size change. After controlling for covariate effects, each species-specific model included a common residual trend that showed overall decline in salmon size across time (Supplementary Fig. S6). This result suggests that salmon might be responding to one or more physical or biological drivers that were not included among the environmental covariates explored.

Metabolic effects of temperature on size^26^ do not appear to be driving body size changes in Alaska salmon (see Supplementary Methods section “Metabolic effects of temperature on size”). Relationships between salmon body size and temperature did not fit the predictions of the metabolic theory of ecology^26^. Rather, the variable influence of climate drivers suggests that the impact of climate on salmon body size is species-specific and to a lesser extent location-specific (see Supplementary Fig. S4), perhaps occurring through climate-mediated changes in food availability or quality. A similarly variable relationship between temperature and body size across species was recently uncovered in a large-scale analysis of size trends in Australian reef fishes^27^.

Due to limited data availability, we investigated the effects of average harvest rate on long-term body length change in a separate analysis on the subset of populations for which we had sufficient harvest information. We expected that if fisheries-induced size structure truncation, or evolution, contributed to size declines, populations subjected to higher rates of size-selective harvest would show greater magnitude declines^28^. We tested this hypothesis using 33 populations (25 sockeye and eight Chinook) with sufficient data to rigorously calculate harvest rate. Counter to expectations, we detected no significant relationship between harvest rate and change in body size among populations (Supplementary Fig. S5, *R*^2^ = 0.02, *F*_1,30_ = 0.56, *p* = 0.46).

### Consequences of declining body size

**Fig. 5:**
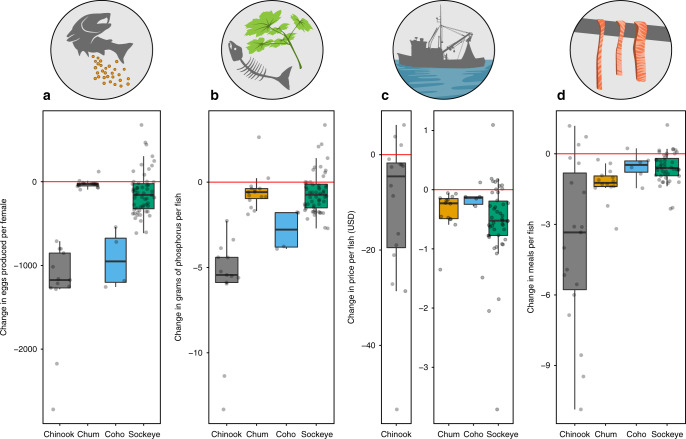
Size declines could result in negative consequences for ecosystems and people. Salmon body size declines over the past 30 years have negative consequences for **a** fecundity, **b** nutrient transport, **c** commercial fishery value, and **d** rural food security. We estimated the difference in ecosystem services provided by an average salmon before 1990 versus after 2010, by converting change in mass to change in services provided. A meal is the species-specific average reported meal size in grams reported by subsistence users from two villages in nearby Yukon Territory, Canada, see Methods for details. Each gray point represents an estimate for an individual population. The red line represents no change in ecosystems services provided by each fish. Center line represents the median, box limits represent the upper and lower quantiles, whiskers represent the 1.5× interquartile range. Sample sizes are presented in Supplementary Data 4.

To quantify the per-capita change in several ecosystem services resulting from observed declines in body size, we used species-specific length-weight relationships to convert change in length to change in mass (see Methods for details). Next, we converted change in mass to per-capita changes in fecundity, nutrient transport, human nutrition, and commercial value (Fig. 5). The per-capita effects of size declines will be most impactful when accompanied by decreases in abundance, as observed for Chinook salmon, whose abundances^23^ and body sizes have both declined in recent years. Our estimates suggest that the dramatic body size declines observed in Chinook salmon translate to equally dramatically reduced per-capita contributions to people and nature, including median reductions in egg production (−15%), commercial value (−25%), meals provided (−26%), and nutrient transport (−26%). Reductions for other species were less dramatic, but still substantial (Fig. 5, Supplementary Data 1–3).

## Discussion

We provide comprehensive evidence that four species of Pacific salmon in Alaska are now smaller than they were historically, with the rate of decline having accelerated since the year 2000. Declining body size overwhelmingly results from younger maturation (i.e., age-at-return) rather than reductions in growth (i.e., size-at-age). Although no single factor explained size declines, we revealed that both climate and competition at sea are associated with changes in salmon size across Alaska. This result extends the findings of other recent studies that also show impacts of climate and competition on salmon body size^20^ and age-at-maturity^29^. Finally, we show that declines in body size over the past 30 years have likely translated into important ecological and socioeconomic consequences for salmon-dependent ecosystems and peoples in Alaska, especially for the largest of the species, Chinook salmon.

Widespread declines in body size occurred over the past four decades across four salmon species (Fig. 1, Fig. 2a). This finding generalizes previous species- and region-specific analyses^19,30,31^. Size trends were more similar for a given species across regions than for a given region across species (Fig. 1), with Chinook salmon showing the greatest decline in size (−8.0%), followed by coho salmon (−3.3%), chum (−2.4%) and sockeye (−2.1%). In contrast to many previous studies that assume monotonic linear changes in size^18,19^, our use of general additive models revealed markedly nonlinear changes, including an apparent recent acceleration of size decline beginning around 2000 that was shared among all four species, and several common periods of high and low average size among sockeye, chum, and coho salmon (Fig. 2a). Identifying the putative drivers of specific periods of time exhibiting shared body size change was beyond our scope, but is likely a fruitful avenue for future research.

Underlying the general body size decline observed across species, a considerable amount of among-region and among-population variation in body size change was observed within species. Body size trends were best explained by models that allowed region-specific (chum and sockeye salmon) or population-specific (Chinook and coho salmon) responses through time, rather than a single response shared among regions and populations (Supplementary Table S1). We interpret this result to reflect the large number of populations sampled from diverse habitats across Alaska, from temperate rainforest ecosystems in Southeast Alaska to subarctic ecosystems in Kotzebue. The idiosyncratic responses of body size to climate indices we observed could be partially explained by differential responses across species, regions, and populations according to site-specific habitat climate filtering, evolutionary histories, and relative location in their species range or climate envelope.

To an unknown extent, other external factors likely also contributed to variation in patterns of size declines among regions and species. For example, the relatively low magnitude body size declines in Southeast Alaska Chinook salmon (Fig. 1) could be explained by an unusual characteristic of the Southeast Alaska troll fishery for Chinook salmon, which catches a high proportion of immature salmon from British Columbia, Washington, Oregon, and California^32^. Reductions in the size and age of Chinook salmon originating from these areas outside of Alaska have not been as extreme as those observed for Alaskan Chinook salmon populations^20,31^.

Earlier maturation (age-at-return), rather than slower growth (size-at-age), was primarily responsible for observed size declines across species and regions (Fig. 3). Chinook salmon, which exhibit the greatest life history diversity and thus greatest capacity for change in age-at-maturity, showed the greatest magnitude of decline in both body size and age-at-maturity. This result formalizes and extends findings from previous studies that age truncation appears to play an important role in declining Chinook salmon body size^19,30,31,33^. Compared to Chinook salmon, changes in age-at-maturity were more variable through time in chum and sockeye salmon (Fig. 2), which also showed size declines but of lower magnitude. Both chum and sockeye salmon showed an initial increase in average saltwater age, but this increase has been followed by generally decreasing age-at-maturity, coinciding with the pronounced recent declines in body size.

Although our results provide strong evidence that salmon are becoming smaller because they are returning from the ocean at a younger age, we were unable to distinguish the contributions of changing maturation schedules from increasing marine mortality. Younger age structure could result from numerous scenarios, including plastic responses to positive growth conditions that allow salmon to reach a threshold size earlier^34^, evolutionary shifts in maturation schedules^35^, increased late-stage mortality^36^, compounding risk from overall increased mortality^36^, or any combination of the above. Finer-scale information about marine mortality is needed to explore these non-mutually exclusive scenarios. It is also important to recognize that the potential for growth rate to influence age-at-maturity^34^ means that, despite the lesser contributions of changing size-at-age, some proportion of the changes in age-at-maturity that contribute to body size declines might ultimately result from changes in growth rate.

Climate and competition at sea clearly influence salmon size. Results for each species indicated a strong effect of at least one climate metric. However, specific metrics varied in their direction and magnitude across species, underscoring the complex effects of climate on body size (Fig. 4). Recent work on salmon productivity has shown that relationships between salmon and climate variables vary through time^37^, and the influence of climate on body size could be similarly non-stationary.

Competition metrics also had important but variable effects on salmon body size (Fig. 4; Supplementary Fig. S4). The strongest negative association we detected was between sockeye salmon body size and the North Pacific-wide abundance of pink salmon. This result corroborates previous studies documenting negative influences of Asian pink salmon abundance on Alaskan sockeye salmon, which share similar prey communities and distributions during their final years at sea^38^. Indeed, the only consistently negative effect across all species was that of Alaskan pink salmon abundance (Fig. 4), although this effect was weak in most species. Intriguingly, the shared acceleration of size declines post-2000 occurred during a period of unusually high (though variable) pink salmon abundance in Alaska^39^, suggesting high pink salmon abundances could be accelerating or exacerbating size declines. Our results provide further evidence that wild and hatchery-enhanced pink salmon abundance in the North Pacific has reached such high levels that they appear to be exerting an influence on ecosystem structure and function^40^.

For each species, we detected an underlying trend shared among populations (i.e., a nonlinear year effect) that was not fully explained by any climate or competition covariates (Supplementary Fig. S6). These shared trends suggest that, within species, populations are responding similarly to other broad-scale factors we did not identify as a priori hypotheses and as a result were not included in our models.

Our results are consistent with previous studies that suggest fisheries are likely not a major driver in broad patterns of salmon size decline^20,29,41^, yet might play an important role for some populations^42,43^. Harvest has been implicated in size and age declines for many marine fishes^5,28^ and has long been expected to contribute to declining salmon size^17^. We did not detect any overall relationship between harvest rate and size change, but our analysis was necessarily limited to a subset of intensively monitored Chinook and sockeye salmon populations with adequate data. Furthermore, the potential for differences in size selectivity across fisheries and gear types^44^ could limit the extent to which these results can be extrapolated to other fisheries.

We lacked sufficient data to investigate several factors that could contribute to size declines, especially in certain species or regions. In Alaska, there is relatively little contribution of hatchery production to the overall abundances of sockeye, coho, and Chinook salmon^29,39^, but hatchery selection^45^ could contribute to size declines in regions with high hatchery production, such as chum salmon in Prince William Sound and Southeast Alaska. We were unable to rigorously test for an effect of hatchery selection, but populations from hatchery-intensive regions did not appear to show greater magnitude declines in body size compared to populations from other regions (Fig. 1). We also lacked sufficient data on predator abundances to test for effects of size-selective predation, but bioenergetic modeling has shown that size-selective predation from killer whales (*Orcinus orca*)^41^ and salmon sharks (*Lamna ditropis*)^46^ could be contributing to body size declines in Chinook salmon. The limited diet data available for Alaska resident killer whales^47,48^ suggests that they show lower selectivity on Chinook salmon than do killer whales from Washington and British Columbia^49^ upon which these models are based^41^. Additional data on hatchery selection, predator abundances, selectivity for salmon, and size-selectivity are needed in Alaska in order to rigorously test these hypotheses.

We estimate that the observed salmon size declines could already be causing substantial reductions in fecundity, nutrient transport, economic value, and food security (Fig. 5). Declines in fecundity can impede population productivity and recovery^50^. Due to these effects on productivity, declines in body size have been used in other systems to predict population declines and collapses^51^. Reduced salmon size also decreases the per-capita transport of marine-derived nutrients into terrestrial ecosystems, with important implications for a wide array of ecological processes including riparian productivity and biodiversity^15^. Salmon are economically important; in 2017, the ex-vessel value (price paid to fishermen) of Bristol Bay salmon fisheries alone was over $214,000,000^52^. Meanwhile, the value of subsistence salmon fisheries for rural and Indigenous communities is profound, with broad implications for food security, well-being, and cultural connectivity^13,14^. Socioeconomic impacts of declining salmon size have long been of concern for Alaskans, especially those whose well-being, food security, and economic livelihoods depend on salmon^14^.

We considered per-capita delivery of ecosystem services, but the realized consequences of declining body sizes will also depend on salmon abundances. The consequences of declining size could, to some extent, be balanced by increasing abundances in some species such as sockeye and chum salmon whose abundances have generally increased in recent years throughout the state^39^. In contrast, Chinook salmon abundances have generally declined across Alaska^23^, so the socioeconomic impacts of declining Chinook salmon size are already compounded by reduced abundance and resulting regulatory limitations on harvest opportunity. Because Alaska salmon are managed according to a fixed escapement policy under which the number of adult salmon that reach the spawning grounds is held generally constant across years, increases in total abundance tend to result in large harvests but generally do not translate into increased escapement. The relatively stable numbers of salmon on the spawning grounds, even in years of high abundance, will result in limited ability for high abundances to mitigate the per-capita ecological consequences of declining size. How increasing salmon abundance might offset the costs of declining body size for the commercial fishery is a complex topic worthy of further exploration, especially for sockeye and chum salmon.

We also acknowledge that other external factors will impact the consequences of declining body size. For example, the economic costs of declining body size are also influenced by idiosyncrasies of production costs and market fluctuations due to trade policies or the availability of market substitutes like farmed Atlantic salmon^53^. These complexities are extremely difficult to fully address at a state-wide multispecies level, but in-depth species-specific considerations of the potential consequences of size declines that account for abundance are important topics for future investigation.

Our findings contribute to the mounting body of evidence that maintenance of body size, in addition to abundance, is critical for maintaining healthy salmon-people and salmon-ecosystem relationships. Yet, what are the options to slow or even reverse these size declines? While the impacts of size declines are experienced locally, the primary causes appear to be regional and even global. Of the two primary drivers associated with size declines, climate forcing and ocean abundance of salmon and particularly Alaska pink salmon, the latter is within local management control. Across the Pacific Rim, ca. 5 billion hatchery salmon^39^ are released into the North Pacific each year where they add to already high abundances of wild pink, chum, and sockeye. While signals of conspecific and interspecific competition are increasingly evident^38,40,54,55^, managers currently lack tools to help inform difficult decisions regarding hatchery releases. Tools that quantify the apparent trade-offs between the releases of one species and the impacts of size and productivity on conspecifics and other species are urgently needed.

Our large-scale consideration of salmon body size extends and generalizes previous findings, showing that body size declines are ongoing and more widespread than previously reported. The direct relationship between smaller salmon and economic and social losses has not been estimated previously. Our conservative calculations of the potential per-capita consequences of recent body size declines show the ecological, economic, and social losses could be substantial. We compared current size to a pre-1990 baseline, but this captures only a small window of commercial salmon fisheries in Alaska, which started in the late 1800s. Size declines were observed long before 1990^17^, and thus we expect that analyses over longer time series would likely reveal even more dramatic impacts. Despite widespread reporting of body size declines across diverse taxa^2,3^, the ecological and socioeconomic consequences of body size declines are underappreciated. Using Pacific salmon in one of the few remaining intact, largely pristine salmon ecosystems on Earth as a test case, we show the consequences for people and ecosystems could be substantial.

## Methods

### Age-length (AL) datasets

Alaska Department of Fish & Game (ADF&G) monitors the number, body size, sex, and age of Alaska salmon harvested in a variety of fisheries and on their return breeding migration from the ocean to freshwater. Age and body length (AL) data have been collected on mature adults from commercial, subsistence, and sport harvests, escapement (spawning population) projects, and test fisheries since the early 1900’s. ADF&G data has historically been archived in regional offices; however, for this project we were able to compile all available data from across the state (Supplementary Figs. S7–S10) into a single dataset, representing over 14 million raw AL samples.

The majority of Alaska salmon fisheries target mature adults during their breeding migration into freshwater. Data from commercial harvests represent the largest proportion (57%) of measurements and are generally collected from marine waters and near river mouths. Although many Alaska salmon fishing districts are designed to operate as terminal fisheries, targeting fish destined for their river of origin, even terminal fisheries can intercept salmon returning to other Alaskan populations, and many other districts are non-terminal. Because most commercial salmon fisheries in Alaska catch a combination of fish from the target stock and intercepted fish returning to other populations, commercial samples often include a mix of fish from different populations within a river drainage and outside the drainage (e.g., Southeast Alaska troll fishery may be >80% non-local fish at times). Commercial samples from some fisheries targeting wild salmon could include a relatively low but unknown proportion of hatchery-origin salmon, which could not be excluded from our analyses without individual-level information on origin (hatchery or wild). Samples from escapement enumeration projects (sampling projects that count the number of mature adults that ‘escape’ the fishery and return to freshwater) make up the next highest proportion of AL measurements (33%). Escapement projects collect AL data from fish sampled in the freshwater environment, close to or on the spawning grounds, generally at counting towers, weirs, or fences. A variety of other sampling project types (test fishing, subsistence catch, sport catch) make up the remaining portion of these data, with no single project type representing more than 5% of the samples. ADF&G recorded the name of the sampling project, generally as the name of a given river (e.g., Fish Creek) or district (e.g., Togiak District), which we refer to as sampling locations. To ensure as much as possible that methods of data collection were consistent across locations and species, we excluded data collected from projects other than commercial harvest and escapement monitoring from statistical analyses.

Age and length (AL) measurements were collected by ADF&G personnel using standard methods^56^. Briefly, fish length is collected to the nearest millimeter using a measuring tape or a manual or electronic measuring board, depending on project and year. Fish age was most commonly estimated by ADF&G scientists reading growth annuli on scales^57^. For many AL measurements, specimen sex was also recorded, predominantly using external characteristics for sex determination. Sex determination with external characteristics in ocean-phase fish is frequently unreliable^58^. Because most of our data come from commercial harvests that occur in ocean-phase fish prior to the development of obvious external secondary sexual characteristics, we did not analyze the sexes separately. However, other studies examining length at age with reliable sex determination have shown similar trends in size and age for males and females^33,59^. As in Lewis et al.^19^, we assume our results reflect similar trends in male and female salmon.

To ensure data were of high quality, a number of quality assurance checks were established, and data failing those checks were excluded from analysis. These checks include ensuring that ages and lengths were within reasonable bounds for each species, that sample dates were reasonable, that data were not duplicated, and that data were all of the same length measurement type (mid-eye to fork of tail). Because mid-eye to fork length was by far the most commonly used length measurement type (85% of samples) within the data, and the vast majority of sample protocols use mid-eye to fork measurements, we assumed that observations where no length measurement type was reported (0.08% of samples) were mid-eye to fork. No other unique length measurement type accounts for more than 2% of samples. We also excluded any samples that measured fewer than ten fish for a given year/location combination. After these extensive checks, we were left with measurements on over 12.5 million individual salmon.

A wide variety of gear types were used to collect samples. The three most common gear types included gillnet, seine, and weir. Sampling methods within projects did not change systematically over time; however, for at least some projects, changes did occur, such as changes in gillnet mesh materials and sizes (for commercial harvest^60^) or sampling location within a watershed (for escapement projects). Some of these methodology changes are sporadically reflected in the data (e.g., mesh size), whereas others are not included and difficult to capture (e.g., weir location changes). Given the inconsistency in data and metadata associated with these fine-scale methodology changes, and the spatial and temporal scale of this dataset, changes in mesh size, gear type, or fine scale location changes (movement of a project within the same river system) were not included in our analyses.

### Consistency in salmon size declines

To quantify the spatial and temporal extent of body size change, we estimated the average length of fish for each species in each sampling location and return year (the year when the fish was caught or sampled on its return migration to freshwater), which we interpret as putative biological populations (henceforth referred to as populations). For each population, we averaged these annual means to find the mean body length during a baseline period before 1990 and recent period after 2010. The pre-1990 period included all data collected before 1990, though relatively little data was available before 1980. Comparing data from two discrete time periods avoids potential edge effects that would be introduced in dividing a consecutive time series. Only populations for which we had data in both periods were included (100 sockeye, 34 Chinook, 32 chum, and 13 coho salmon populations). We established a criterion of at least 3 years of data for each population during each time period for inclusion in this analysis. Although somewhat arbitrary, we chose 1990 as the end of the early period to ensure a large number of populations had sufficient data to be included, while still being early enough to provide a meaningful baseline for comparison with current data. Because our goal was to investigate trends experienced by resource users in Alaska, we included data from some stocks that are known to capture salmon that originated from areas other than Alaska. For example, estimates for Chinook salmon from Southeast Alaska are likely influenced by the inclusion of troll-caught Chinook salmon, which are largely composed of salmon originating from British Columbia (B.C.) and the U.S. West Coast. For visualization, the results of this analysis were then scaled up to the level of the fisheries management areas established by ADF&G (Fig. 1).

To quantify and visualize continuous changes in body size across time, we fit general additive models (GAMs) to annual mean population body length for each species. To avoid convergence problems due to small sample sizes, data collected before 1975 were excluded from this analysis. In contrast to previous studies that assumed monotonic linear changes in size^18,19^, year was included as a nonlinear smoothed term because preliminary analyses suggested that the rate of length change varied through time. We included data from all populations for which observations from five or more years were available (276 sockeye salmon populations, 202 Chinook salmon populations, 183 chum salmon populations, 142 coho salmon populations). We knew a priori that salmon populations differ in average body size, so to preserve original units (mm) while controlling for variation in absolute body length among populations, we included two fixed factors: population and region. We assigned regions based on terrestrial biomes and the drainage areas of major watershed (shown numbered on Fig. 1, colored by ADF&G management region). Repeating these GAMs on escapement data alone provided equivalent results (Supplementary Fig. S11), which confirms that our results are not due to an artifact of sampling procedures through time.

To visualize changes in age structure and size-at-age, we fit very similar GAMs to age and length-at-age data. As above we included fixed effects for population and region, as well as a nonlinear year effect. Using the same dataset as the previously described GAMs, we used either mean freshwater age, mean saltwater age, or mean length-at-age as the response variable. For length-at-age, we separately fit GAMs for the four most common age classes in each species, except coho salmon, for which sufficient data was available for only three age classes.

To determine the extent to which patterns of body size change are consistent across space within a species, we re-fit these GAMs by replacing the main year effect by either a region-by-year or population-by-year interaction and compared model fit using AIC. These nonlinear interactions allow regions or populations to differ in their patterns of length change through time. These models are more data intensive than the previous GAMs, so we included data from all populations for which our time series consisted of any 20 or more years of data (123 sockeye salmon populations, 37 Chinook salmon populations, 38 chum salmon populations, 14 coho salmon populations).

### Contributions of declining age versus growth

To partition the contribution of changes in population age structure versus size-at-age to changes in mean population length, we used the chain rule^61^. We used the discrete time analog of the chain rule

1 $${\Delta}\left( {xy} \right) = y{\Delta}x + x{\Delta}y,$$

and assume that change in mean length is a function of changes in population age structure, *p(a*), and mean length-at-age, *x(a)*. For each species and population, age structure in year *t* was calculated as the proportion of individuals in each age *a*. Mean length in year *t* is given by

2 $$x_t = {\Sigma}_ap_t\left( a \right)x_t\left( a \right),$$

and the year-to-year change in length is given by

3 $${\Delta}x_t = x_{\left( {t + 1} \right)} - x_t = {\Sigma}_ap_t\left( a \right)x_t\left( a \right) + {\Delta}p_t\left( a \right)x_t\left( a \right),$$

where

4 $$p_t(a) = 1/2\left[ {p_{t + 1}(a) + p_t(a)} \right],$$

and

5 $${\Delta}p_t(a) = \left[ {p_{t + 1}(a) - p_t(a)} \right].$$

Solving these formulas year-to-year for each species in each population, we estimated the proportion of change in mean length due to changes in age structure and size-at-age. We included all populations for which we had five or more years of data (though change can only be estimated for consecutive years of data) and averaged the results across populations in each region.

### Causes of age and size changes

To identify potential causes of change in salmon body size, we quantified associations with a variety of indices describing physical and biological conditions in Alaska’s freshwater and marine salmon habitats. Each candidate explanatory variable was selected based on existing biological hypotheses or inclusion in previous analyses of salmon size or population dynamics.

We considered several ocean climate indicators as potential causes of change in salmon size over time. Pacific Ocean conditions are often quantified using large-scale climate indices such as the Pacific Decadal Oscillation (PDO), El Niño Southern Oscillation (ENSO), and NPGO. These large-scale indices of ocean conditions, as proxies for climate and marine environment, have been shown to affect the survival and productivity of Pacific salmon in the North Pacific Ocean^62,63^. PDO, NPGO^64^, and MEI^65,66^ indices were all accessed and downloaded online (PDO, http://research.jisao.washington.edu/pdo/; NPGO, http://www.o3d.org/npgo/npgo.php, accessed 2018-02-07; MEI, https://www.esrl.noaa.gov/psd/enso/mei/, accessed 2018-02-08; MEIw, https://www.beringclimate.noaa.gov/, accessed 2018-02-08). In this analysis, winter means of NPGO and MEI were used in addition to an annual mean of MEI. Two ice cover metrics were also used to capture ocean climate conditions. Bering Sea ice cover and retreat were downloaded from https://www.beringclimate.noaa.gov/, originally derived from the National Snow and Ice Data Center data. Bering Sea ice cover index represents the winter anomaly, relative to 1981–2000 mean. Bering Sea ice retreat is an index representing number of days with ice cover after March 15.

Sea surface temperature (SST) was also explored as a potential cause of the changes in salmon size and age. SST has proven to be closely linked to salmon productivity. Mueter et al.^67^ found that regional-scale SST predicted survival rates better than large-scale climate indices such as the PDO. They concluded that survival rates were largely driven by environmental conditions at regional spatial scales. SST was extracted from the Extended Reconstructed Sea Surface Temperature (ERSST) version 4^68^. To approximate SST values close to the river mouths which juvenile salmonids are most likely to experience after ocean entry, a double layer of the grid cells tracing the coastline of Alaska were extracted and the mean summer SST was calculated for each region.

Because in situ fluvial temperature measurements are sparse, both spatially and temporally, compared to the coverage of the AL dataset, air temperature was used as a proxy for temperature during the freshwater life stages. Air temperature data were extracted and sorted from remote-sensed satellite observations into multi-monthly regional means by season^69^.

Finally, we considered the potential for competition with other salmon to influence salmon size by including the abundances of several highly abundant salmon species as explanatory covariates. Using data compiled by Ruggerone and Irvine^39^, we evaluated the abundance of adult pink, chum, and sockeye salmon returning to Asia and North America as a proxy for the abundance of adult salmon of each species in the North Pacific. In addition, we also considered the more localized abundance of pink, chum, and sockeye salmon returning to Alaska, because salmon body size has been shown to vary with salmon abundance in the year of return migration in some species^70^ at finer spatial scales. The abundances of coho and Chinook salmon were not included, because they occur at much lower abundance than sockeye, chum, and pink salmon.

We also explored marine mammal abundances as potential predictor variables, but found that the data available precluded rigorous statistical comparison with our time series of salmon size and age structure. For example, the only estimates of orca abundance available for our study area (that from Southeast Alaska and Prince William Sound) show steady, near monotonic increases through our study period^71,72^. Statistically, this leads to insufficient replication and high collinearity with year effects. Although caution is warranted in interpretations of any models for which the assumptions are so obviously violated, we note that preliminary analyses including marine mammal abundance were not dramatically superior in terms of variance explained or model fit. Because of these limitations, we determined that a reliable test of the effect of marine mammal predation was not possible for Alaska.

Ultimately, we only selected covariates with an absolute correlation among covariate time series of less than 0.61. By establishing this threshold for absolute pairwise covariate correlation we sought to include only covariates for which separate associations with salmon size could be identified. The final set of covariates included in our analyses were: (1) ocean climate indicators (PDO, NPGO, MEI, winter MEI (MEIw), and Bering Sea ice cover index); (2) sea surface temperature (SST); (3) air temperature as proxy for freshwater temperature; and (4) ocean salmon abundance (abundance of Alaska sockeye, pink, and chum salmon, and North Pacific wide abundance of sockeye, pink, and chum salmon).

To test hypothesized associations between temporal trends in the average body size (length) of salmon and environmental conditions, we fit a series of Bayesian hierarchical models to data describing size trends across sampling locations for each species. Because the chain rule analysis showed that changes in age structure explained greater interannual body size variation than did changes in size-at-age, we analyzed age-aggregated mean body length. Time series, starting in 1975, of annual mean length by species for each sampling location (*l*) and environmental covariates were mean-variance (*Z*) standardized prior to model fitting. Models of the form

6 $$L_{i,t} = \mathop {{\Sigma}}\limits_c ( {\beta _{l,c} * X_{t - \delta _{c,}c}} ) + s\left( t \right) + \varepsilon _{l,t},$$

were fit to each salmon species separately using Bayesian methods, where *L*_*l,t*_ is the standardized length at each location (*l*) in each return or observation year (*t*), *β*_*l,c*_ are coefficients describing the effect of each covariate (*c*) on average length at each location, and $X_{t - \delta _{c,}c}$ is the standardized value of each covariate in each year. The reference year for each covariate is specified relative to the return year, or year in which salmon length compositions are observed (*t*), by a species and covariate-specific offset *δ*_c_ that associates covariate effects with the hypothesized period of interaction in each species’ life history (Supplementary Table S2). Location-specific covariate effects are structured hierarchically such that parameters describing the effect of each covariate on observed changes in average length were subject to a normally-distributed prior whose hyperparameters (group-level means and standard deviations for each covariate) were estimated directly from the data:

7 $$\beta _{l,c} \sim {\mathrm{Normal}}\left( {\mu _c,\tau _c ^{2}} \right),$$

This hierarchical structure permitted us to quantify both the average (group-level) association between length observations at each sampling location (*l*) and hypothesized covariates (i.e., the hyperparameter *μ*_c_), and the level of among-location variation in these effects (i.e., $τ_c^{2}$). Prior distributions for model parameters were generally uninformative, with the exception of the prior on the group-level mean covariate effects (*μ*_c_) which included a mild penalty toward zero,

8 $$\mu _c \sim {\mathrm{Normal}}\left( {0,1} \right).$$

The prior distribution of the group-level (hyper) standard deviation of covariate effects was broad and truncated at zero,

9 $$\tau _c \sim {\mathrm{Normal}}\left( {0,10} \right)\left[ {0,} \right],$$

allowing the model to freely estimate the appropriate level of among-location variability in covariate effects.

Observation error was assumed to be normally distributed *ε*_*l,t*_ *~* *Normal(0, σ*_ε_^2^*)*, with a common observation error variance (*σ*_*ε*_^2^) estimated as a free parameter and subject to a broad prior distribution

10 $$\sigma _\varepsilon \sim {\mathrm{Normal}}\left( {0,10} \right)\left[ {0,} \right].$$

Each species-specific model also included a smoothed nonlinear year effect *s(t)* describing residual trends in length across time that were shared among sampling (observation) locations but were not explained by the covariates. The degree of nonlinearity for the univariate smooth *s(t)* quantifying the common residual trend in length is controlled by the variance term (*σ*_*s*_) for the coefficients forming the spline^73^, for which a broad zero-truncated prior distribution was defined:

11 $$\sigma _s \sim {\mathrm{Normal}}\left( {0,10} \right)\left[ {0,} \right].$$

Hierarchical Bayesian models describing the temporal trend in location-specific salmon length were fit using the brms package^73,74^ in R (R Core Team 2018), which generates posterior samples using the No U-Turn Sampler implemented in the Stan software platform^75^. Three independent chains were run for 20,000 iterations with a 50% burn-in and saving every tenth posterior sample, resulting in 3000 posterior samples. Convergence of all chains was diagnosed by ensuring potential scale reduction factors (*R̂*) for each parameter were <1.05^76^. The sensitivity of model results to prior choice was evaluated by testing more and less restrictive normally-distributed priors for the hyperparameters describing the group-level average effect of each covariate (standard deviation 1.0 and 0.1); estimated covariate effects were insensitive to prior choice.

The influence of harvest on body size was considered separately from that of climate and competition. Reviews of fisheries-induced evolution have shown that populations subject to higher harvest rates show greater magnitude trait change^28^, thus we expected that if fisheries-induced evolution contributes to size change, populations subjected on average to higher harvest rates should show greater magnitude negative size change. To test this hypothesis, we estimated harvest rate as a continuous variable for all populations with sufficient data.

Harvest rate was back-calculated from brood tables, which are datasets curated by ADF&G for management purposes that include the number of offspring from each brood year (year of birth) that return in each of the subsequent years (return year). Brood tables are only available for the most intensively managed salmon stocks. We were able to link brood table data to populations included in our AL datasets for 25 sockeye salmon populations and three Chinook salmon populations. Harvest rates were found from the literature for an additional five Chinook salmon populations^77–79^. To calculate the total harvest in each population and year, we subtracted escapement estimates from the overall estimate of returns (i.e., total run size, or both fish that escaped and were harvested). Harvest rate was calculated as the harvest divided by the estimated run size in each year, then averaged across the time series for each population to obtain the average harvest rate experienced by each salmon population. Averaging across the time series was deemed appropriate, because previous studies from the few Alaska salmon fisheries with sufficient data to consider harvest rate through time have shown that harvest rate is interannually variable but relatively stable through time^33,60^. Estimates from before 1990 or after 2010 (for sockeye) or 2008 (for Chinook) were excluded due to incomplete data availability. Each population for which both a brood table and AL data were available had a long time series of AL data (at least 30 years), so body size change was calculated by fitting a linear model of body length by year and extracting the slope. We regressed change in body size (slope coefficient of length-year regression) against population-specific harvest rate averaged through time (1990–2012), with a fixed effect for species. A harvest rate by species interaction was included but removed because it was not significant. *P* values were obtained from an ANOVA with type II sum of squares.

### Consequences of declining body size

To estimate the potential consequences of salmon body size declines, we calculated the change in ecosystem services that would be expected given the observed change in body length for several important social, economic, and ecological roles filled by salmon in Alaska. For each species and population, we calculated percent change in body size (body length, Δ*L*) from pre-1990 to post-2010 using the same methods as described for Fig. 1. Specifically, we calculated absolute change in body size as:

12 $${\Delta}L = {\mathrm{Mean}}\,{\mathrm{length}}_{{\mathrm{post}} - 2010} - {\mathrm{Mean}}\,{\mathrm{length}}_{{\mathrm{pre}} - 1990},$$

and percent change in body size as:

13 $${\mathrm{Percent}}\,{\mathrm{size}}\,{\mathrm{change}} = \frac{{{\mathrm{Mean}}\,{\mathrm{length}}_{{\mathrm{post}} - 2010} - {\mathrm{Mean}}\,{\mathrm{length}}_{{\mathrm{pre}} - 1990}}}{{{\mathrm{Mean}}\,{\mathrm{length}}_{{\mathrm{pre}} - 1990}}}.$$

However, the magnitude of many of the ecosystem services we investigated vary with salmon body mass, rather than directly with body length. To predict salmon weight (*W*) based on body length (*L*), we fit a standard length–weight relationship of the form *W* = *a*(*L*)^*b*^. Weight data were not available for most regions, so we estimated the *a* and *b* parameters for each species by fitting the logarithmic linearized version of this equation to high-quality datasets collected in Alaska for each species (Supplementary Table S3). Using these species-specific length-weight relationships, for each species and location, we calculated the change in weight between 1990 and 2010 (Δ*W*) by finding the weight of an average post-2010 salmon and subtracting the weight of an average pre-1990 salmon. Detailed results are presented in Supplementary Data 1–3.

To consider the ecological consequences of salmon body size change, we focused on data collected by “escapement projects”. These projects usually sample salmon in-river at a weir or counting tower as they migrate upstream onto spawning grounds. For each location with sufficient data (three or more years in each time window, before 1990 and after 2010), we estimated the ecological consequences of salmon size decline as the change in marine-derived phosphorus transported and the change in the number of eggs produced per fish. To calculate change in phosphorus inputs, we modified previously-developed models for anadromous fish nutrient loading to include only the import of nutrients into fresh waters by spawning adults^80,81^. We used a previously-estimated phosphorus content for spawning adult salmon of 0.38% of wet weight^80,81^. We calculated the difference in phosphorus content using the mean weight before 1990 versus after 2010. We ignored the effect of juvenile export on nutrient loading due to insufficient data and because previous studies have found its effect to be negligible unless adult biomass and escapement are extremely low^81^.

To calculate the change in female fecundity, we used fecundity–length relationships to estimate the fecundity of the average female before 1990 and after 2010 and found the difference. We used published, species-specific fecundity–length relationships estimated for populations within Alaska. Because fecundity data were not available for all regions, we based these relationships on high-quality datasets from representative populations within Alaska (Supplementary Table S4).

To consider the economic consequences of body size change, we focused on data sampled from commercial fisheries. For each location with sufficient data (three or more years in each time window), we asked how much higher per-fish ex-vessel prices would be if fish had not changed in size in the period between 1990 and 2010. That is, using current price-per-pound estimates, we compared the price of two fish: one that weighed the same as an average fish post-2010 and one that weighed the same as the average fish pre-1990. First, we identified the most recently reported ex-vessel prices for each species and region^82^. For each species and region, we then multiplied the weight of the average pre-1990 salmon by its corresponding price-per-pound to calculate the average ex-vessel price for a pre-1990s salmon in today’s market. This value was then subtracted from the average ex-vessel value of a post-2010 salmon, calculated in the same way, to estimate the change in ex-vessel per-capita salmon value due to salmon size change.

To consider the social consequences of size change, we focused on data from salmon caught in subsistence fisheries. However, length measurements taken from subsistence projects were rarely available before 1990. For this reason, we also included data from salmon caught in commercial harvest, which are expected to use the most similar gear types (i.e., gillnets) to subsistence harvest. For each location with sufficient subsistence or commercial data (three or more years in each time window), we modeled the social consequences of salmon size decline as the change in nutrient content and total servings or meals per fish. First, we determined the change in edible mass (*M*) of each fish by scaling according to species-specific values for seafood processing recovery rates^83^. We assumed that subsistence recovery rates are similar to the reported recovery rates for hand-filleted skin-on fillets, which were 55% for Chinook salmon, 60% for chum salmon, 57% for coho salmon, and 53% for sockeye salmon. We expect fillets to be the most commonly used salmon part but acknowledge that subsistence users could use different body parts (including the head and eyes) and that true recovery rates will likely vary among locations and users. We then calculated the nutrient value of the average pre-1990 and post-2010 fish and calculated the change in nutrient value, using species-specific nutritional ratios for protein (g), fat (g), and calories (kcal) per 100 g serving^84^. We used nutritional ratios for raw fish (National Nutrient Database for Standard Reference IDs: 15,078 for Chinook, 15,081 for coho, 15,085 for sockeye, and 15,079 for chum salmon). We also asked how many fewer 100 g servings and how many fewer meals of salmon were available per fish. We assume a standard serving size of 100 g, but note that many individuals will eat more than one serving in a sitting. Because of this uncertainty in serving size, we also included the change in meals by dividing M by the average self-reported estimates of portion sizes of salmon (227 g for Chinook salmon, 165.5 g for chum salmon, 178 g for coho salmon, and 163.5 g for sockeye salmon) from subsistence users in the nearby villages of Old Crow and Teslin, Yukon Territory, Canada^85^.

### Reporting summary

Further information on research design is available in the Nature Research Reporting Summary linked to this article.

## Supplementary information

Supplementary Information

Reporting Summary

Description of Additional Supplementary Files

Supplementary Data 1

Supplementary Data 2

Supplementary Data 3

Supplementary Data 4

Supplementary Data 5

Supplementary Data 6

Supplementary Data 7

## Data Availability

Our data have been publicly archived on the Knowledge Network for Biocomplexity (KNB): Jeanette Clark, Rich Brenner, and Bert Lewis. 2018. Compiled age, sex, and length data for Alaskan salmon, 1922–2017. Knowledge Network for Biocomplexity. 10.5063/F1707ZTM. Krista B Oke, Curry Cunningham, and Peter Westley. 2020. Collated dataset of covariates that could influence body size of Alaska salmon. Knowledge Network for Biocomplexity. 10.5063/F1N29V9T. In addition, we used publically available data from the following sources: US Department of Agriculture (USDA), Agricultural Research Service Laboratory. USDA National Nutrient Database for Standard Reference, Legacy Version. Available at: http://www.ars.usda.gov/nutrientdata. Alaska Department of Fish and Game. Commercial Salmon Fishery Exvessel Prices by Area and Species (2018). Available at: https://www.adfg.alaska.gov/index.cfm?adfg=commercialbyfisherysalmon.salmoncatch_exvessel (Accessed: 2018-04-23). Kibele, J. & Jones, L. Historic air temperatures in Alaska for 1901–2015, with spatial subsetting by region. (2017). 10.5063/F1RX997V. Huang, B. et al. Extended Reconstructed Sea Surface Temperature (ERSST), Version 4. Accessed on April 16, 2018 (2015). 10.7289/V5KD1VVF. Di Lorenzo et al., 2008: North Pacific Gyre Oscillation links ocean climate and ecosystem change, GRL. Available at: http://www.o3d.org/npgo/npgo.php (Accessed: 2018-02-08). NOAA, Multivariate ENSO Index. Available at: https://www.esrl.noaa.gov/psd/enso/mei/ (Accessed: 2018-02-08). JISAO, Pacific Decadal Oscillation (PDO). Available at: http://www.research.jisao.washington.edu/pdo/ (Accessed: 2018-02-08). NOAA, Bering Sea Ice Cover Index. Available at: beringclimate.noaa.gov (Accessed: 2018-02-08). NOAA, Winter Multivariate ENSO Index. Available at: https://www.beringclimate.noaa.gov/data/BCresult.php (Accessed: 2018-02-08).

## Code availability

Code has been archived publicly and is available at: https://github.com/KristaOke/salmon-size-declines.

## References

[1] Peters, R. H. *The Ecological Implications of Body Size*. (Cambridge University Press, 1983).

[2] Gardner, J. L., Peters, A., Kearney, M. R., Joseph, L. & Heinsohn, R. Declining body size: a third universal response to warming? *Trends Ecol. Evol*. **26**, 285–291 (2011). 10.1016/j.tree.2011.03.005 21470708

[3] Sheridan JA, Bickford D (2011). Shrinking body size as an ecologial response to climate change. Nat. Clim. Chang..

[4] Hsieh C, Yamauchi A, Nakazawa T, Wang WF (2010). Fishing effects on age and spatial structures undermine population stability of fishes. Aquat. Sci..

[5] Allendorf FW, Hard JJ (2009). Human-induced evolution caused by unnatural selection through harvest of wild animals. Proc. Natl Acad. Sci. USA.

[6] Ozgul A (2009). The dynamics of phenotypic change and the shrinking sheep of St. Kilda. Science.

[7] Daufresne M, Lengfellner K, Sommer U (2009). Global warming benefits the small in aquatic ecosystems. Proc. Natl Acad. Sci. USA.

[8] Buskirk JVan, Mulvihill RS, Leberman RC (2010). Declining body sizes in North American birds associated with climate change. Oikos.

[9] Law R (2000). Fishing, selection, and phenotypic evolution. ICES J. Mar. Sci..

[10] Olsen EM (2004). Maturation trends indicative of rapid evolution preceded the collapse of northern cod. Nature.

[11] Planque B (2010). How does fishing alter marine populations and ecosystems sensitivity to climate?. J. Mar. Syst..

[12] Audzijonyte A, Kuparinen A, Gorton R, Fulton EA (2013). Ecological consequences of body size decline in harvested fish species: positive feedback loops in trophic interactions amplify human impact. Biol. Lett..

[13] Nesbitt HK, Moore JW (2016). Species and population diversity in Pacific salmon fisheries underpin indigenous food security. J. Appl. Ecol..

[14] Moncrieff, C. How People of the Yukon River Value Salmon: a case study in the lower, middle, and upper portions of the Yukon River. (Final report to the North Pacific Research Board project #1413. Yukon River Drainage Fisheries Association. Anchorage, Alaska, 2017).

[15] Hocking MD, Reynolds JD (2011). Impacts of salmon on riparian plant diversity. Science.

[16] Gustafson RG (2007). Pacific salmon extinctions: quantifying lost and remaining diversity. Conserv. Biol..

[17] Ricker WE (1981). Changes in the average size and average age of Pacific salmon. Can. J. Fish. Aquat. Sci..

[18] Bigler BS, Welch DW, Helle JH (1996). A review of size trends among North Pacific salmon (*Oncorhynchus* spp.).. Can. J. Fish. Aquat. Sci..

[19] Lewis B, Grant WS, Brenner RE, Hamazaki T (2015). Changes in size and age of chinook salmon *Oncorhynchus tshawytscha* returning to Alaska. PLoS ONE.

[20] Jeffrey KM, Côté IM, Irvine JR, Reynolds JD (2016). Changes in body size of Canadian Pacific salmon over six decades. Can. J. Fish. Aquat. Sci..

[21] Steen RP, Quinn TP (1999). Egg burial depth by sockeye salmon (*Oncorhynchus nerka*): implications for survival of embryos and natural selection on female body size. Can. J. Zool..

[22] Carlson SM, Quinn TP, Hendry AP (2011). Eco-evolutionary dynamics in Pacific salmon. Heredity.

[23] Ohlberger J, Scheuerell MD, Schindler DE (2016). Population coherence and environmental impacts across spatial scales: a case study of Chinook salmon. Ecosphere.

[24] Hunt LM (2005). Recreational fishing site choice models: insights and future opportunities. Hum. Dimens. Wildl..

[25] Gelman A, Goodrich B, Gabry J, Vehtari A (2019). R-squared for Bayesian regression models. Am. Stat..

[26] Munch SB, Salinas S (2009). Latitudinal variation in lifespan within species is explained by the metabolic theory of ecology. Proc. Natl Acad. Sci. USA.

[27] Audzijonyte A (2020). Fish body sizes change with temperature but not all species shrink with warming. Nat. Ecol. Evol..

[28] Sharpe DMT, Hendry AP (2009). Life history change in commercially exploited fish stocks: an analysis of trends across studies. Evol. Appl..

[29] Cline TJ, Ohlberger J, Schindler DE (2019). Effects of warming climate and competition in the ocean for life-histories of Pacific salmon. Nat. Ecol. Evol..

[30] McPhee MV, Leon JM, Wilson LI, Siegel JE, Agler BA (2016). Changing growth and maturity in western Alaskan Chinook Salmon *Oncorhynchus tshawytscha*, brood years 1975–2005. North Pac. Anadromous Fish. Comm. Bull..

[31] Ohlberger J, Ward EJ, Schindler DE, Lewis B (2018). Demographic changes in Chinook salmon across the Northeast Pacific Ocean. Fish. Fish..

[32] Shelton AO, Satterthwaite WH, Ward EJ, Feist BE, Burke. B (2018). Using hierarchical models to estimate stock-specific and seasonal variation in ocean distribution, survivorship, and aggregate abundance of fall run Chinook salmon. Can. J. Fish. Aquat. Sci..

[33] Kendall NW, Quinn TP (2011). Length and age trends of Chinook salmon in the Nushagak River, Alaska, related to commercial and recreational fishery selection and exploitation. Trans. Am. Fish. Soc..

[34] Thorpe JE (2007). Maturation responses of salmonids to changing developmental opportunities. Mar. Ecol. Prog. Ser..

[35] Czorlich Y, Aykanat T, Erkinaro J, Orell P, Primmer CR (2018). Rapid sex-specific evolution of age at maturity is shaped by genetic architecture in Atlantic salmon. Nat. Ecol. Evol..

[36] Hard JJ (2008). Evolutionary consequences of fishing and their implications for salmon. Evol. Appl..

[37] Litzow MA (2018). Non-stationary climate–salmon relationships in the Gulf of Alaska. Proc. R. Soc. B Biol. Sci..

[38] Ruggerone GT, Connors BM (2015). Productivity and life history of sockeye salmon in relation to competition with pink and sockeye salmon in the North Pacific Ocean. Can. J. Fish. Aquat. Sci..

[39] Ruggerone GT, Irvine JR (2018). Numbers and biomass of natural- and hatchery-origin Pink Salmon, Chum Salmon, and Sockeye Salmon in the North Pacific Ocean, 1925–2015. Mar. Coast. Fish. Dyn. Manag. Ecosyst. Sci..

[40] Springer AM, van Vliet GB (2014). Climate change, pink salmon, and the nexus between bottom-up and top-down forcing in the subarctic Pacific Ocean and Bering Sea. Proc. Natl Acad. Sci. USA.

[41] Ohlberger J, Schindler DE, Ward EJ, Walsworth TE, Essington TE (2019). Resurgence of an apex marine predator and the decline in prey body size. Proc. Natl Acad. Sci. USA.

[42] Bromaghin JF, Nielson RM, Hard JJ (2011). A model of Chinook salmon population dynamics incorporating size-selective exploitation and inheritance of polygenic correlated traits. Nat. Resour. Model..

[43] Kendall NW, Dieckmann U, Heino M, Punt AE, Quinn TP (2014). Evolution of age and length at maturation of Alaskan salmon under size-selective harvest. Evol. Appl..

[44] Kendall NW, Quinn TP (2012). Quantifying and comparing size selectivity among Alaskan sockeye salmon fisheries. Ecol. Appl..

[45] Grant WS (2012). Understanding the adaptive consequences of hatchery-wild interactions in Alaska salmon. Environ. Biol. Fishes.

[46] Manishin, K. A. Exploring the potential role of late stage predation and Chinook salmon age structure. (University of Alaska Fairbanks, 2018).

[47] Saulitis EL, Matkin CO, Barrett-Lennard L, Heise K, Ellis GM (2000). Foraging strategies of sympatric killer whale (*Orcinus orca*) populations in Prince William Sound, Alaska. Mar. Mammal. Sci..

[48] Herman DP (2005). Feeding ecology of eastern North Pacific killer whales *Orcinus orca* from fatty acid, stable isotope, and organochlorine analyses of blubber biopsies. Mar. Ecol. Prog. Ser..

[49] Ford JKB, Ellis GM (2006). Selective foraging by fish-eating killer whales *Orcinus orca* in British Columbia. Mar. Ecol. Prog. Ser..

[50] Walsh MR, Munch SB, Chiba S, Conover DO (2006). Maladaptive changes in multiple traits caused by fishing: impediments to population recovery. Ecol. Lett..

[51] Clements CF, Ozgul A (2016). Including trait-based early warning signals helps predict population collapse. Nat. Commun..

[52] Prepared by the McDowell Group for the Bristol Bay Regional Seafood Development Association. *2017 Sockeye Market Analysis*. (2017).

[53] Valderrama D, Anderson JL (2010). Market interactions between aquaculture and common-property fisheries: recent evidence from the Bristol Bay sockeye salmon fishery in Alaska. J. Environ. Econ. Manag..

[54] Springer AM (2018). Transhemispheric ecosystem disservices of pink salmon in a Pacific Ocean macrosystem. Proc. Natl Acad. Sci. USA.

[55] Ruggerone GT, Nielsen JL (2004). Evidence for competitive dominance of Pink salmon (*Oncorhynchus gorbuscha*) over other Salmonids in the North Pacific Ocean. Rev. Fish. Biol. Fish..

[56] Brenner, R. & Moffitt, S. *Operational Plan: Salmon Age, Sex, Size and Stock of Origin Sampling from Prince William Sound Area Fisheries and Escapements;* Regional Operational Plan CF.2A.2014.02. (Alaska Department of Fish & Game, Anchorage, Alaska, 2014).

[57] Tobias, T. M., Waltemyer, D. L. & Tarbox, K. E. *Scale Aging Manual for Upper Cook Inlet Sockeye Salmon;* Regional Information Report No. 2A94-36 (Alaska Department of Fish & Game, Anchorage, Alaska, 1994).

[58] Brodersen, A. R., Liller, Z. W. & Truesdale, C. L. *Salmon**Age, Sex, and Length Catalog for the Kuskokwim Area, 2012*. (Alaska Department of Fish & Game, Anchorage, Alaska, 2013).

[59] Shaul LD, Geiger HJ (2016). Effects of climate and competition for offshore prey on growth, survival, and reproductive potential of coho salmon in Southeast Alaska. North Pac. Anadromous Fish. Comm. Bull..

[60] Kendall NW, Hard JJ, Quinn TP (2009). Quantifying six decades of fishery selection for size and age at maturity in sockeye salmon. Evol. Appl..

[61] Hairston NG, Ellner SP, Geber MA, Yoshida T, Fox JA (2005). Rapid evolution and the convergence of ecological and evolutionary time. Ecol. Lett..

[62] Mantua NJ, Hare SR, Zhang Y, Wallace JM, Francis RC (1997). A Pacific interdecadal climate oscillation with impacts on salmon production. Bull. Am. Meteorol. Soc..

[63] Hare BSR, Mantua NJ, Francis RC (1996). Inverse production regimes: Alaska and west coast Pacific salmon. Fish. Habitat.

[64] Lorenzo EDI, Mantua N (2016). Multi-year persistence of the 2014/2015 North Pacific marine heatwave. Nat. Clim. Chang..

[65] Wolter K, Timlin MS (1998). Measuring the strength of ENSO events: how does 1997/98 rank?. Weather.

[66] Wolter, K. & Timlin, M. S. Monitoring ENSO in COADS with a seasonally adjusted principal component index. In *Proc. 17th Climate Diagnostics Workshop* 52–57 (1993).

[67] Mueter FJ, Peterman RM, Pyper BJ (2002). Opposite effects of ocean temperature on survival rates of 120 stocks of Pacific salmon (*Oncorhynchus* spp.) in northern and southern areas. Can. J. Fish. Aquat. Sci..

[68] Huang, B. et al. Extended Reconstructed Sea Surface Temperature (ERSST), Version 4. (2015). 10.7289/V5KD1VVF. Accessed 16 April 2018.

[69] Kibele, J. & Jones, L. Historic air temperatures in Alaska for 1901–2015, with spatial subsetting by region. (2017). 10.5063/F1RX997V.

[70] Rogers DE, Ruggerone GT (1993). Factors affecting marine growth of Bristol Bay sockeye salmon. Fish. Res..

[71] Matkin CO, Ward Testa J, Ellis GM, Saulitis EL (2014). Life history and population dynamics of southern Alaska resident killer whales (*Orcinus orca*). Mar. Mammal. Sci..

[72] Chasco BE (2017). Competing tradeoffs between increasing marine mammal predation and fisheries harvest of Chinook salmon. Sci. Rep..

[73] Bürkner P-C (2018). Advanced Bayesian Multilevel Modeling with the R Package brms. R J..

[74] Bürkner P-C (2017). brms: an R package for Bayesian multilevel models using Stan. J. Stat. Softw..

[75] Carpenter B (2017). Stan: a probabilistic programming language. J. Stat. Softw..

[76] Gelman, A., Carlin, J. B., Stern, H. S. & Rubin, D. B. *Bayesian Data Analysis, 2nd edn*. (Texts in Statistical Science, Chapman & Hall, CRC, Boca Raton, USA, 2004).

[77] Mcpherson, S., Clark, J. H., Jones, E., Weller, J. & Ericksen, R. *Stock Status and Escapement Goals for Chinook Salmon Stocks in Southeast Alaska, Special Publication 03-01*. (Alaska Department of Fish and Game, Anchorage, Alaska, 2003).

[78] Piston, A. W. & Heinl, S. C. *Chum Salmon Stock Status and Escapement Goals in Southeast Alaska*. (Alaska Department of Fish and Game, Anchorage, Alaska, 2014).

[79] Bernard, D. R. & Jones, E. L. III *Optimum escapement goals for Chinook salmon in the transboundary Alsek River*, Fishery Manuscript Series No. 10-02. (Alaska Department of Fish and Game, Anchorage, Alaska, 2010).

[80] Moore JW (2011). Nutrient fluxes and the recent collapse of coastal California salmon populations.. Can. J. Fish. Aquat. Sci..

[81] Twining CW, Palkovacs EP, Hasselman DJ, Friedman MA, Post DM (2017). Nutrient loading by anadromous fishes: species-specific contributions and the effects of diversity. Can. J. Fish. Aquat. Sci..

[82] Alaska Department of Fish and Game. Commercial Salmon Fishery Exvessel Prices by Area and Species (2018). www.adfg.alaska.gov/index.cfm?adfg=commercialbyfisherysalmon.salmoncatch_exvessel Accessed 23 April 2018.

[83] Crapo, C., Paust, B. C. & Babbitt, J. *Recoveries and yields from Pacific fish and shellfish*. (2004).

[84] US Department of Agriculture (USDA), Agricultural Research Service Laboratory. USDA National Nutrient Database for Standard Reference, Legacy Version. http://www.ars.usda.gov/nutrientdata.

[85] Schuster RC, Wein EE, Dickson C, Chan HM (2012). Importance of traditional foods for the food security of two First Nations communities in the Yukon, Canada. Int. J. Circumpolar Health.

