# Supplementary material for: Recent declines in salmon body size impact ecosystems and fisheries
Source: Nat Commun. 2020 Aug 19;11:4155. doi: 10.1038/s41467-020-17726-z (PMC7438488; doi:10.1038/s41467-020-17726-z)
Supplement: Supplementary file 1 — Supplementary Information [file 41467_2020_17726_MOESM1_ESM.pdf]

Supplementary Materials for

Recent declines in salmon body size impact ecosystems and fisheries

Oke *et al.*

This PDF file includes:

Supplementary Methods

Supplementary Figures 1-11

Supplementary Tables 1-4

Supplementary methods

Metabolic effects of temperature on size

Reductions in the lifespan and body size of ectotherms in response to increased temperatures have been widely observed and often attributed to the metabolic theory of ecology (MTE;[1]). To determine whether decreases in salmon age could be fully or partially explained by the MTE, we fit our age data to the MTE[2]. For all populations with AL data for 20 or more years after 1970, we regressed the logarithm of average population age against the inverse of temperature in Kelvin:

$$\ln(a) = \text{constant} + \ln(m) + \beta kT \tag{1}$$

where a is age, m is mass, k is Boltzmann's constant, 8.62 x 10^{-5} eV/K, and T is temperature. Munch and Salinas[2] observed that dropping the mass term from the equation did not influence their results and we had very few mass data, so we also dropped the mass term. We fit the equation to both freshwater temperature (using air temperature as a proxy) and nearshore SST at the regional level. In both cases, the data did not meet the expectations of the MTE.

Supplementary Figures

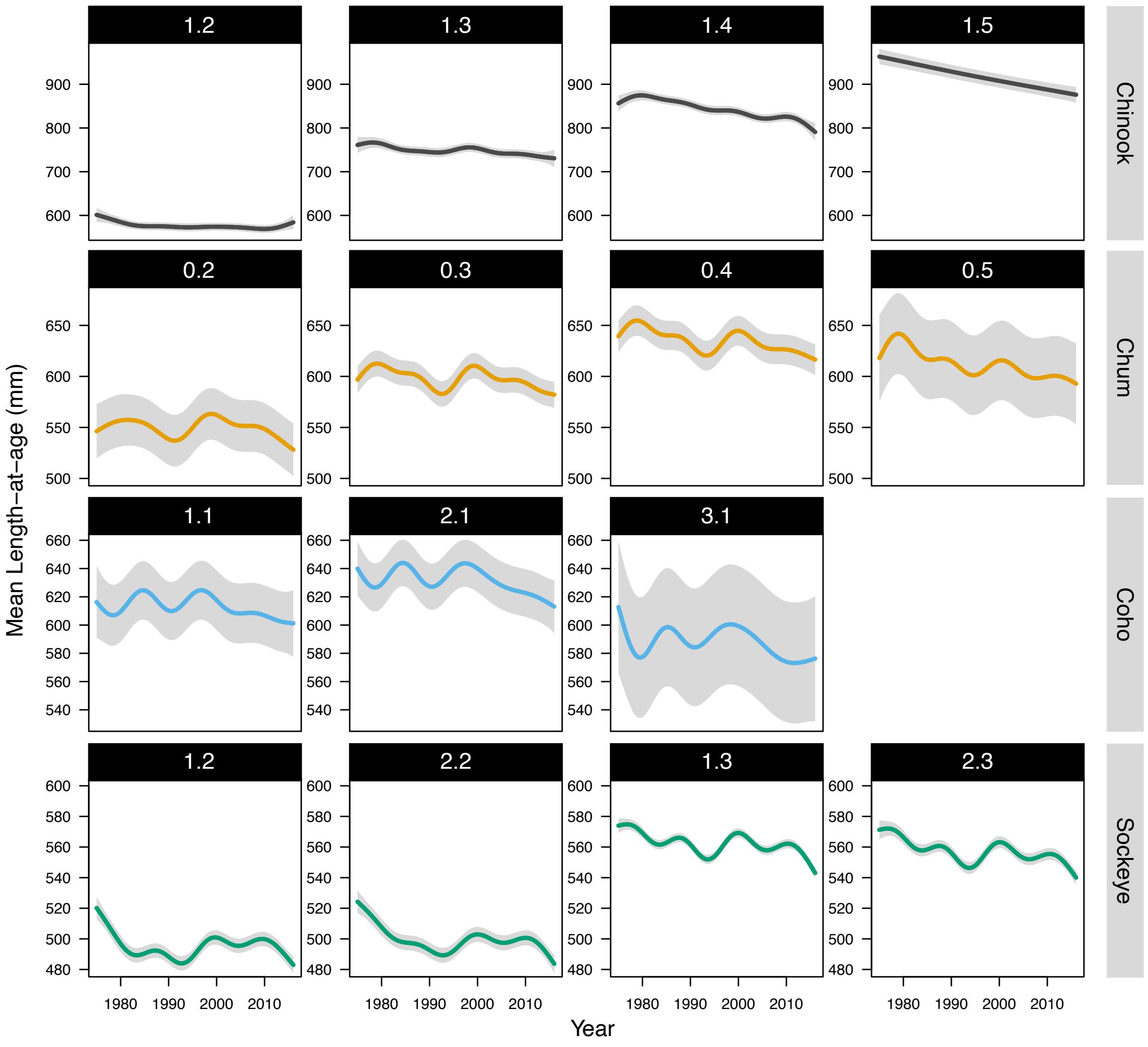

Supplementary Figure 1: Length-at-age has generally declined in Alaska salmon, as shown by the non-linear year effect from GAMs on the population mean length-at-age for the four most common age classes in each species (except coho, for which sufficient data was available for only three age classes). Each panel is labeled by age class following the European system, with fresh water age given first, followed by salt water age. Plots are conditioned on reference populations with the longest time series for each species, but the pattern plotted is the common pattern

through time calculated for all populations. Grey areas represent 95% confidence intervals. Sample sizes are presented in Supplementary Data File 4.

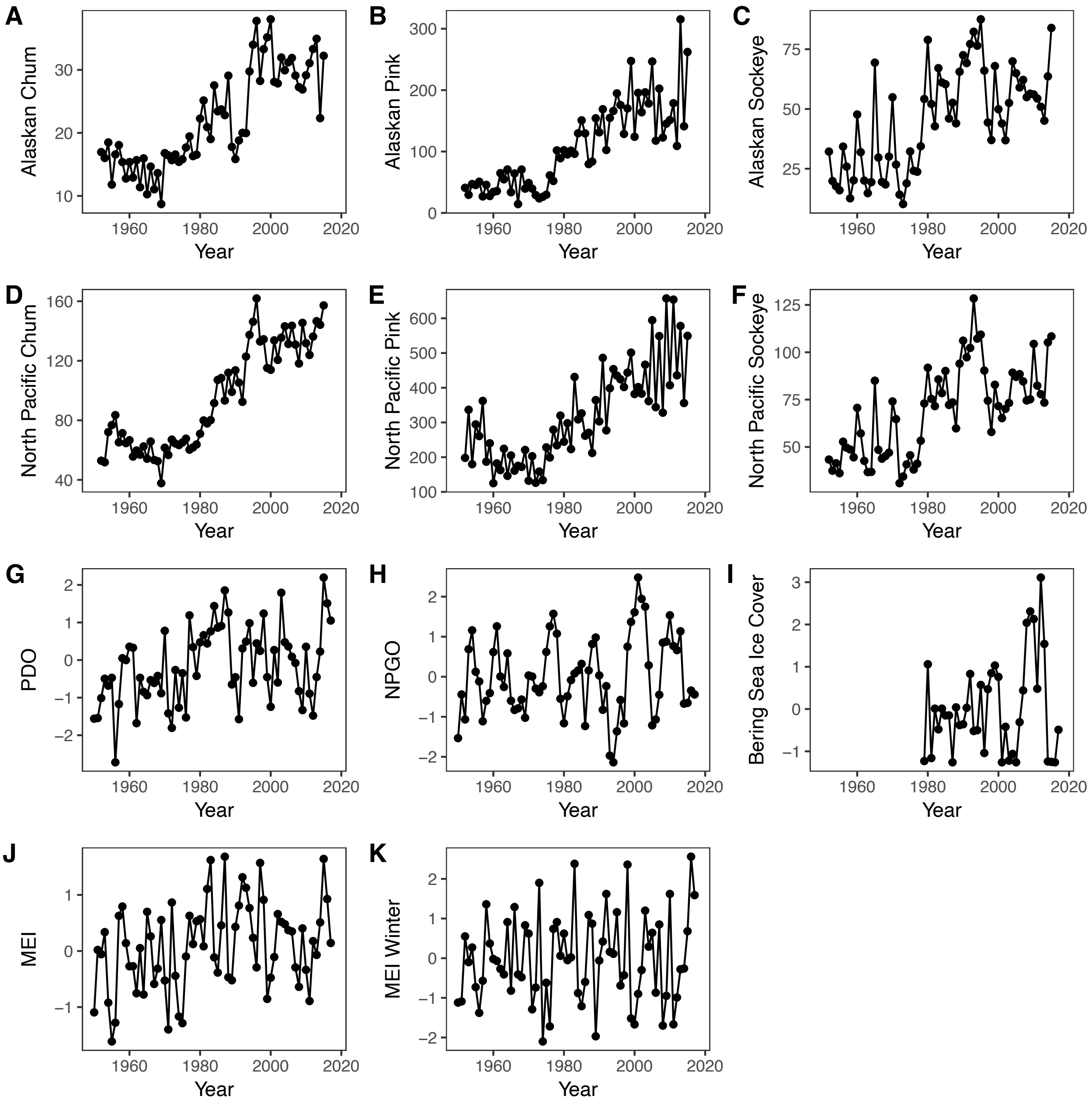

Supplementary Figure 2: Time series of covariates included in our analysis on the influence of climate and competition indices on salmon body size. Mean variance standardized values are presented. For details on how data for each covariate is collected, see Supplementary Methods (section "Causes of salmon size declines").

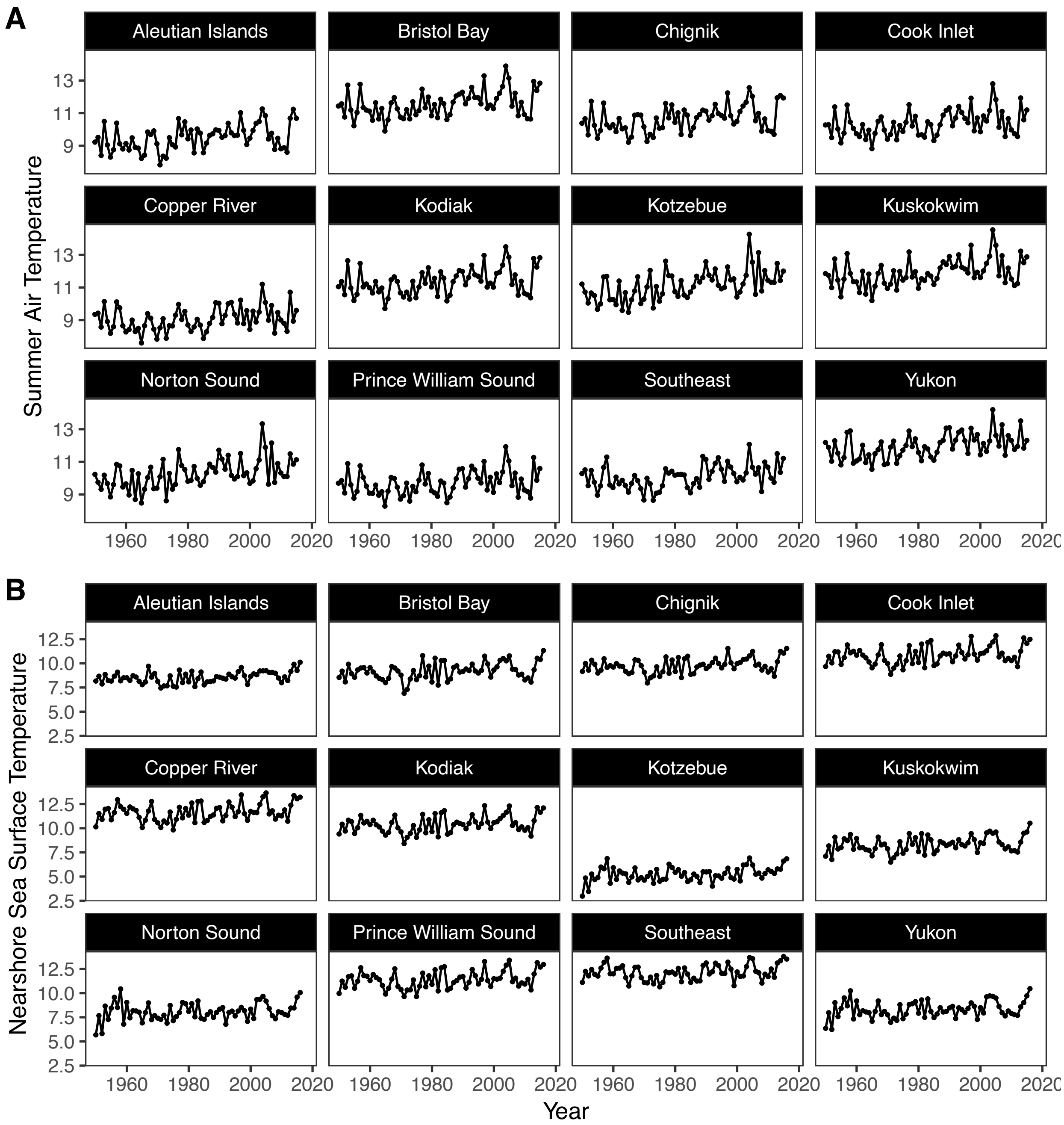

Supplementary Figure 3: Time series of summer air temperature (A) and nearshore sea surface temperature (B) metrics included in our analysis on the influence of climate and competition indices on salmon body size. Mean variance standardized values are presented. For details on how data for each metric was collected, see Supplementary Methods (section "Causes of salmon size declines").

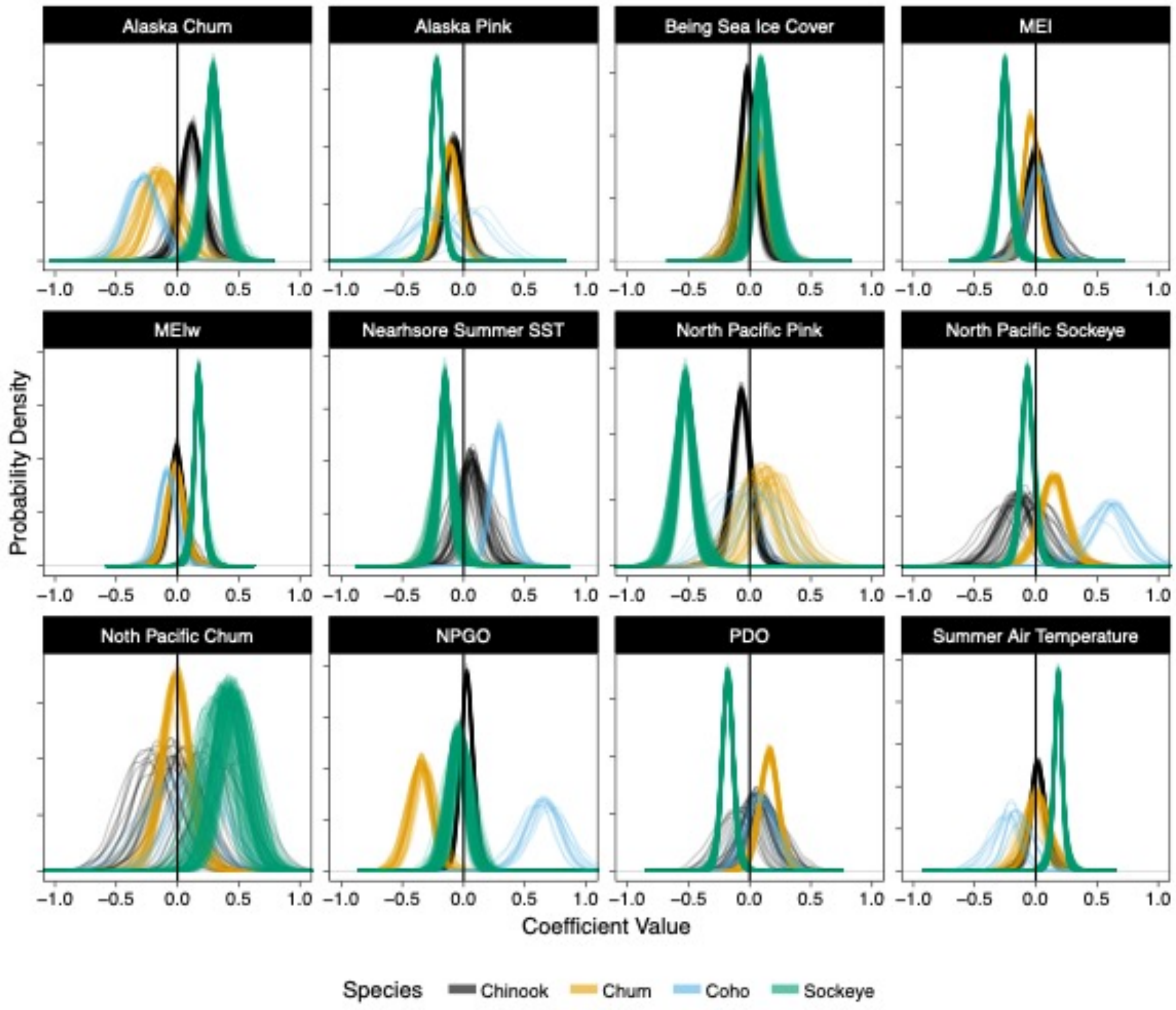

Supplementary Figure 4: The extent of among-population variance varies among the effects of climate and competition metrics. Each colored line describes the posterior probability distribution for the estimated effect of a covariate on a specific population. Population-specific effects for each covariate are colored by species. Sample sizes are presented in Supplementary Data File 7.

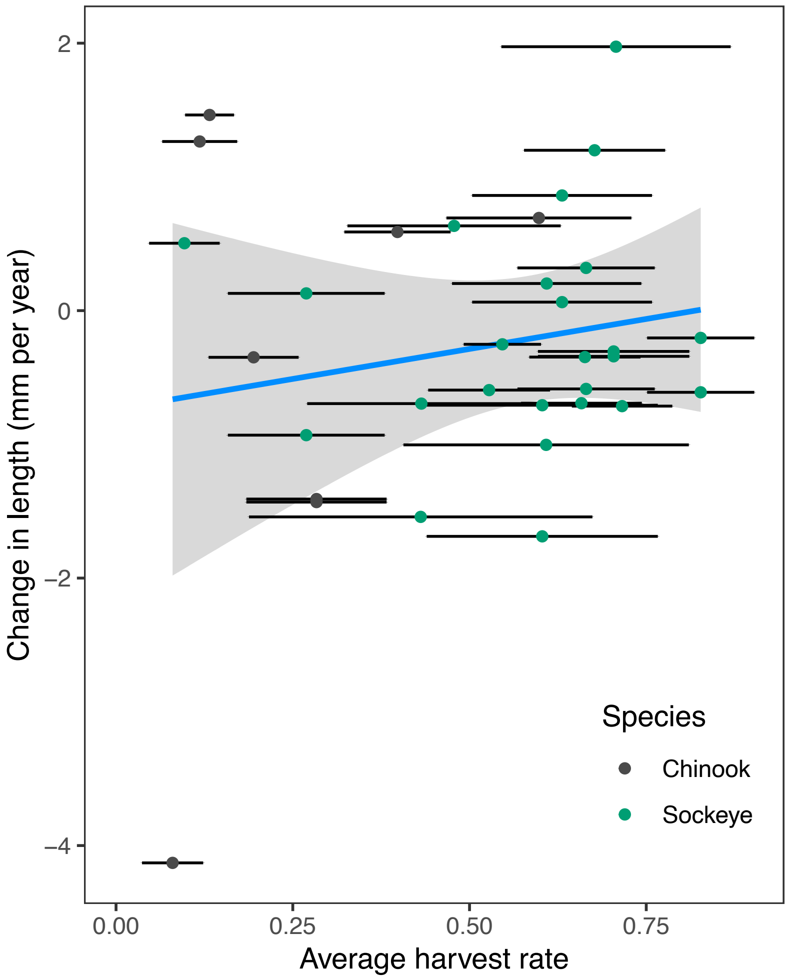

Supplementary Figure 5: We observed no significant relationship between harvest rate and change in body length (shown here as change in mm/year). Grey area represents the 95% confidence interval for the model fit, points represent individual populations (colored by species) with error bars for harvest rate estimates around the mean (±1 standard deviation), and blue line represent the linear model fit to the data. Change in length is based on measurements from 355,896 pre-1990 individuals and 995,333 post-2010 individuals, from 33 populations.

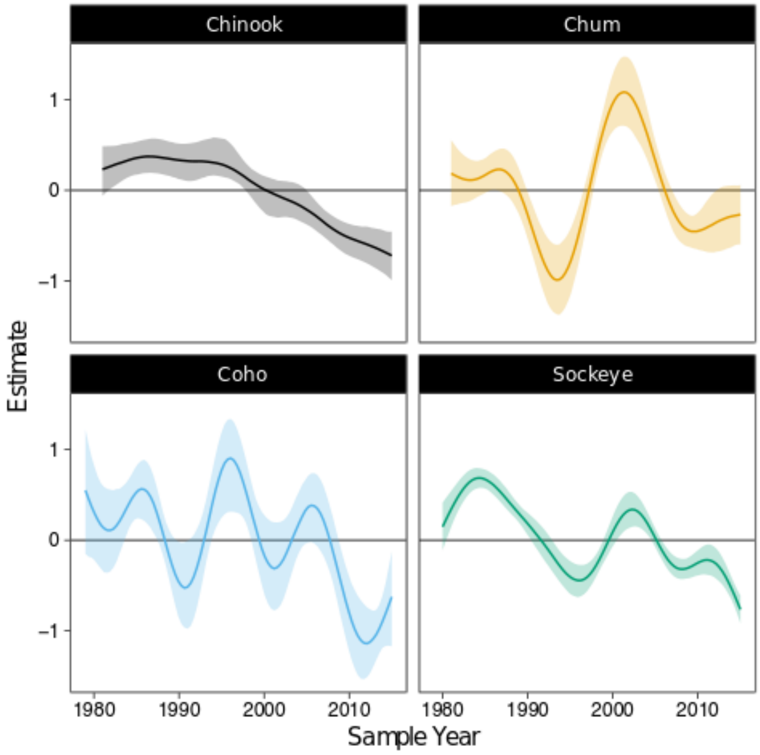

Supplementary Figure 6: The common residual trends (non-linear smoothed year effect, *s(t)*) from each Bayesian model show that for each species, some temporal pattern and recent decline in size was shared among locations that was not explained by any covariate. Shaded areas represent 95% confidence intervals for the residual trend. Sample sizes are presented in Supplementary Data File 7.

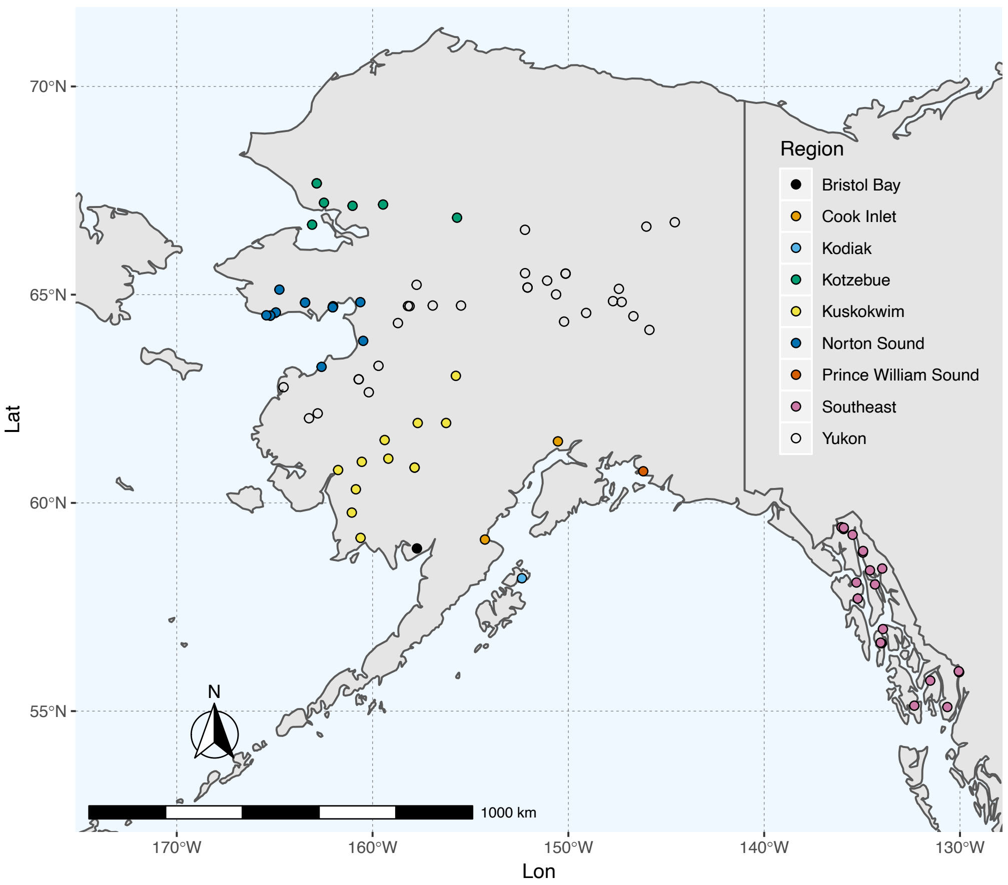

Supplementary Figure 7: Maps of the state of Alaska showing Chinook salmon sampling locations with known coordinates, with sites colored by region. Most escapement projects had known coordinates and are shown, whereas few commercial catch projects had known coordinates.

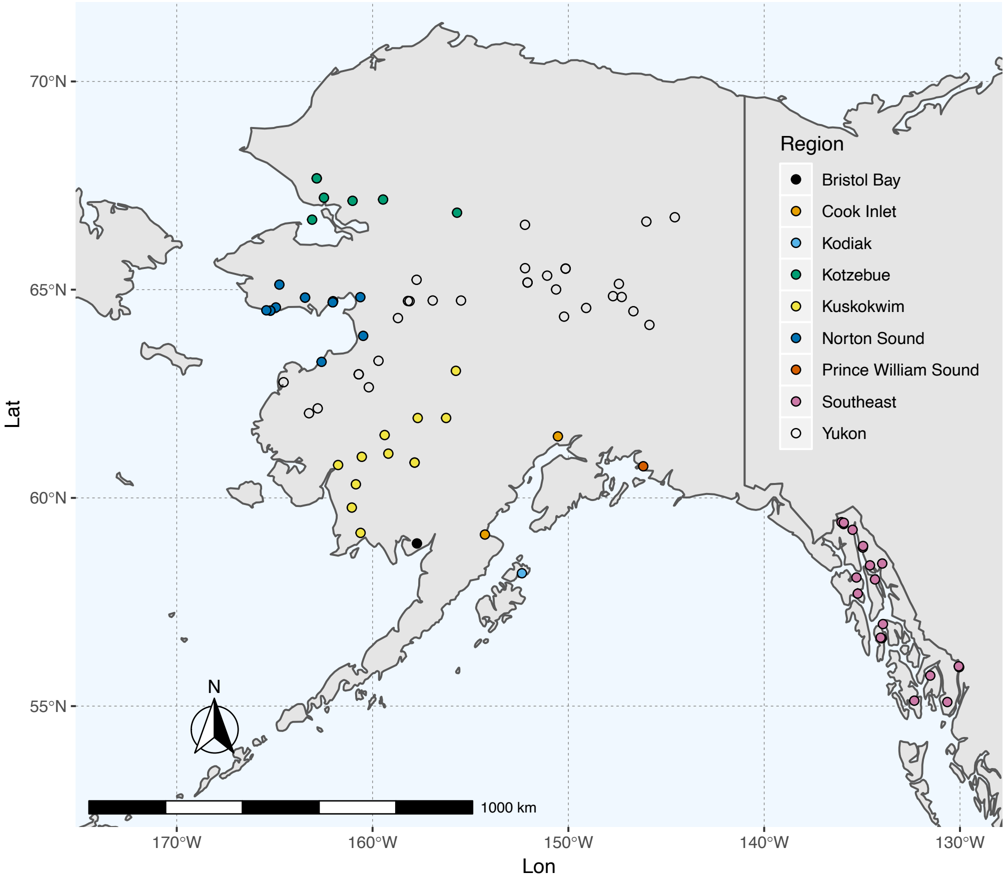

Supplementary Figure 8: Maps of the state of Alaska showing chum salmon sampling locations with known coordinates, with sites colored by region. Most escapement projects had known coordinates and are shown, whereas few commercial catch projects had known coordinates.

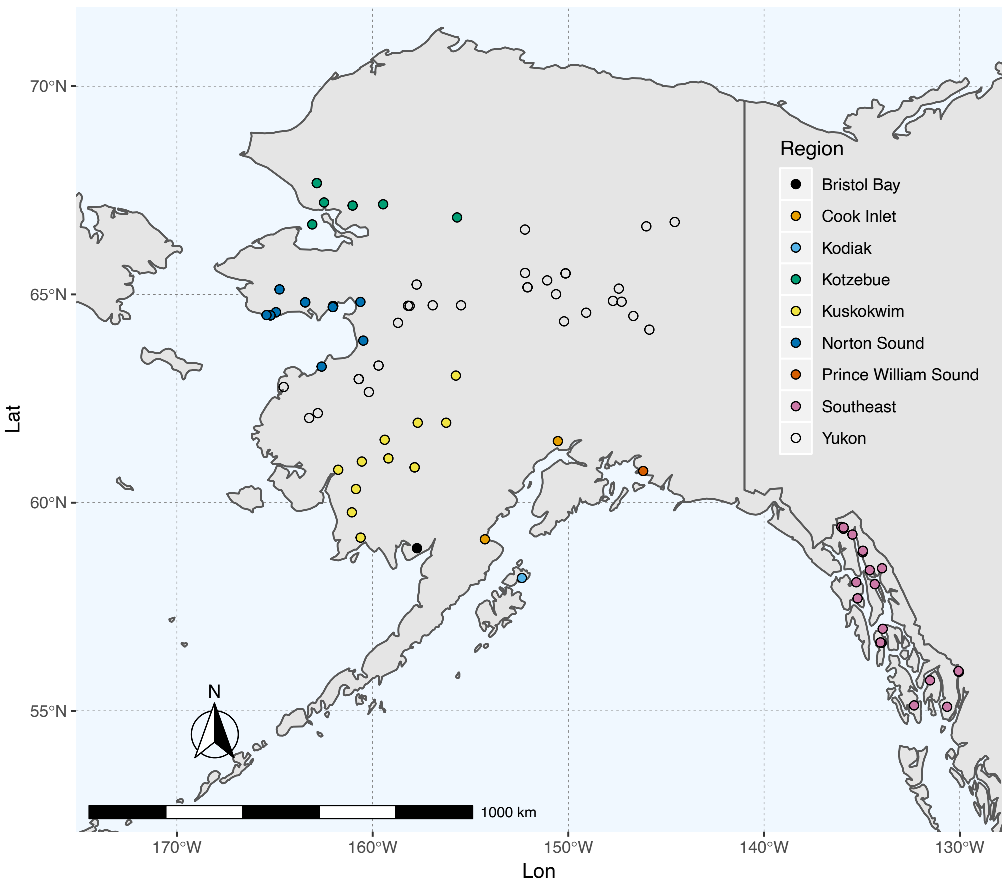

Supplementary Figure 9: Maps of the state of Alaska showing coho salmon sampling locations with known coordinates, with sites colored by region. Most escapement projects had known coordinates and are shown, whereas few commercial catch projects had known coordinates.

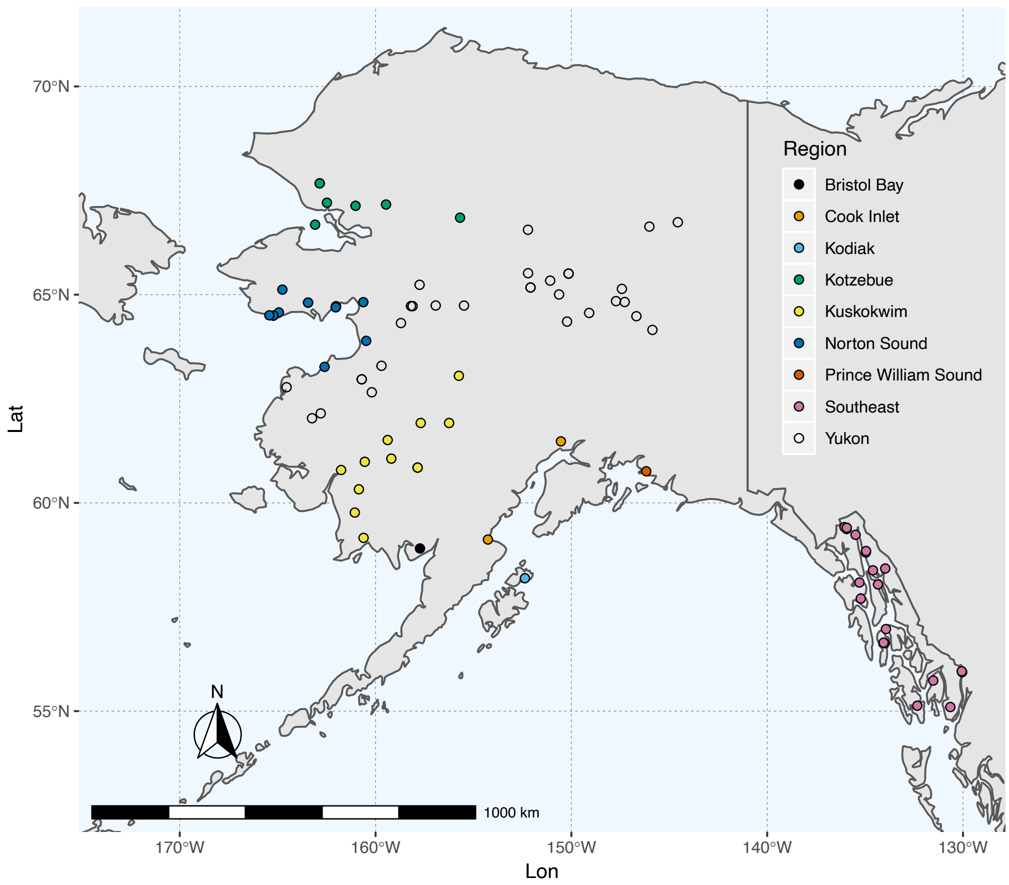

Supplementary Figure 10: Maps of the state of Alaska showing sockeye salmon sampling locations with known coordinates, with sites colored by region. Most escapement projects had known coordinates and are shown, whereas few commercial catch projects had known coordinates.

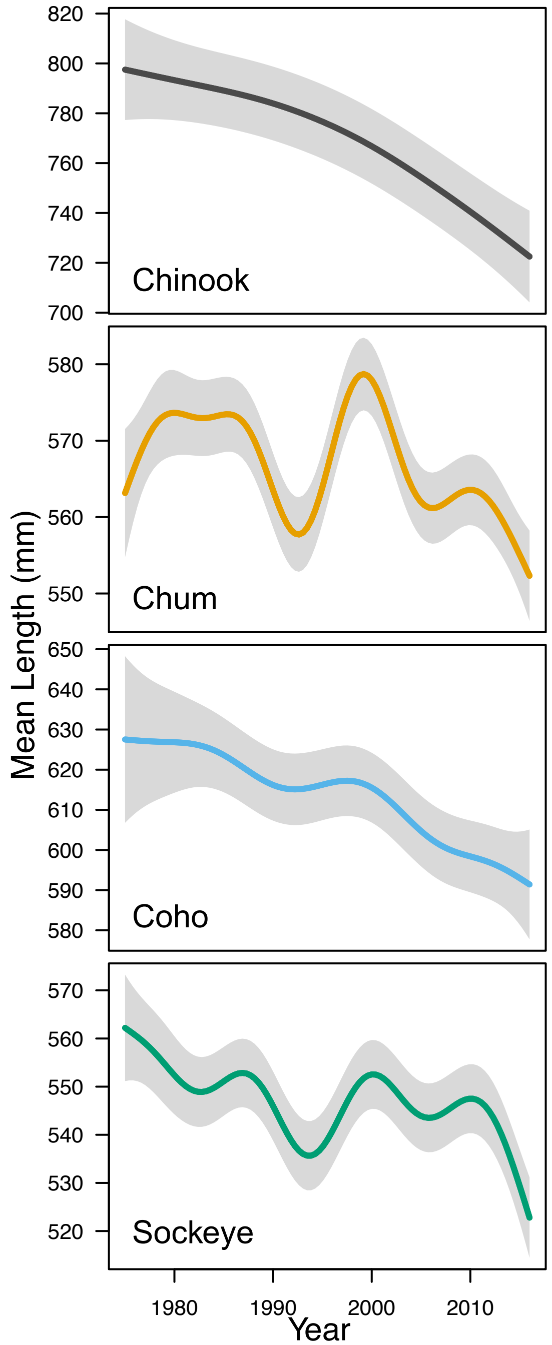

Supplementary Figure 11: Removing data from commercial catch projects to consider mean length through time for escapement samples only results in equivalent results compared to GAMs run on both escapement and commercial catch samples together (See Fig. 2A). Plots are conditioned on reference populations with the longest time series for each species, but the pattern plotted

is the common pattern through time calculated for all populations. Grey areas represent 95% confidence intervals. Sample sizes are presented in Supplementary Data File 4, rows with project type “escapement”.

Supplementary Tables

Supplementary Table 1.

Goodness of fit metrics and statistical results for general additive models of mean body length through time. Models that include a smoothed year effect assume that change in body length has a common pattern among populations and regions. Models that include a region-by-year factor smoothed interaction allow regions to respond differently through time, whereas models that include a population-by-region factor smoothed interaction allow populations to respond differently through time. In the models, ‘bs’ stands for basis spline and ‘fs’ stands for factor smoothed.

Species	model	AIC	Deviance explained	R2	Significance of smoother
Sockeye	standardized mean length ~ s(year)	34750	62.0	0.607	F = 51.00, p = 3.80x10^{-89}
	standardized mean length ~ s(year, region, bs="fs”)	34633	64.2	0.624	F = 7.900, p = 5.27x10^{-98}
	standardized mean length ~ s(year, population, bs="fs")	34898	63.3	0.605	F = 0.412, p = 1.71x10^{-44}
Chinook	standardized mean length ~ s(year)	12763	51.3	0.497	F = 54.42, p = 1.34x10^{-48}
	standardized mean length ~ s(year, region, bs="fs”)	12662	57.9	0.551	F = 4.205, p = 3.27x10^{-38}
	standardized mean length ~ s(year, population, bs="fs")	12621	62.2	0.580	F = 1.706, p = 1.43x10^{-77}
Chum	standardized mean length ~ s(year)	9488	78.4	0.775	F = 32.22, p = 2.27x10^{-52}
	standardized mean length ~ s(year, region, bs="fs”)	9395	82.0	0.802	F = 5.884 p = 5.28x10^{-60}
	standardized mean length ~ s(year, population, bs="fs")	9422	85.4	0.816	F = 2.061, p = 6.78x10^{-55}
Coho	standardized mean length ~ s(year)	3897	75.8	0.746	F = 9.640, p = 2.03x10^{-13}
	standardized mean length ~ s(year, region, bs="fs”)	3891	77.2	0.755	F = 1.702, p = 3.43x10^{-14}
	standardized mean length ~ s(year, population, bs="fs")	3857	79.2	0.774	F = 1.073, p = 1.16x10^{-19}

Supplementary Table 2.

Each covariate was offset from the year of spawning migration by a lag (in years) to ensure that each covariate was tested at a biologically meaningful time

Metric	Lag			
	Chinook	Chum	Coho	Sockeye
PDO	2	2	0	1
NPGO	2	2	0	1
MEI	2	2	0	1
MEIw	2	2	0	1
Bering Sea ice cover	2	2	0	1
Alaska abundance of chum salmon	0	0	0	0
North Pacific abundance of chum salmon	1	0	1	1
Alaska abundance of pink salmon	0	0	0	0
North Pacific abundance of pink salmon	1	1	1	1
North Pacific abundance of sockeye salmon	1	1	1	0
Nearshore summer SST	3	3	1	2
Summer air temperature	4	3	2	3

Supplementary Table 3.

Parameters used in weight-fecundity relationship for each species

Species	Slope	Intercept	Sampling location	Source
Chinook	17.8	-5594	Unalakleet River, Norton Sound	[3]
chum	29.2	766.5	Kwethluk River, George River, Takotna River, Kuskokwim	[4]
coho	22.5	7619	Kodiak	[5]
sockeye	11.7	-2536	Chignik Lake and Black Lake, Chignik	[6]

Supplementary Table 4.

Parameters used in length-weight relationship for each species

Species	*a*	*b*	Sampling location	Source
Chinook	1.77×10^{-9}	3.30	Pilot Station and Eagle, Yukon	[7]
chum	6.30×10^{-9}	3.14	Coghill District, Copper River	unpublished data, ADFG
coho	6.46×10^{-9}	3.14	Copper River District, Copper River	unpublished data, ADFG
sockeye	1.01×10^{-8}	3.10	Copper River, Copper River	unpublished data, ADFG

Supplementary References

1. Brown, J. H., Gillooly, J. F., Allen, A. P., Savage, V. M. & West, G. B. Toward a metabolic theory of ecology. *Ecology* **85,** 1771–1789 (2004).
2. Munch, S. B. & Salinas, S. Latitudinal variation in lifespan within species is explained by the metabolic theory of ecology. *Proc. Natl. Acad. Sci. U. S. A.* **106,** 13860–13864 (2009).
3. Bell, J. & Kent, S. *Chinook salmon fecundity in the Unalakleet River, 2008-2010*. Alaska Department of Fish & Game, Anchorage, Alaska (2012).
4. Gilk, S. E., Templin, W. D., Molyneaux, D. B., Hamazaki, T. & Pawluk, J. A. *Characteristics of Fall Chum Salmon* Oncorhynchus keta *in the Kuskokwim River Drainage*. Alaska Department of Fish & Game, Anchorage, Alaska (2005).
5. Beacham, T. D. Fecundity of coho salmon (*Oncorhynchus kisutch*) and chum salmon (*O. keta)* in the northeast Pacific Ocean. *Can. J. Zool.* **60,** 1463–1469 (1982).
6. Phinney, D. E. & Lechner, J. *Studies of Adult Chignik Sockeye Salmon in 1967*. Alaska Department of Fish & Game, Anchorage, Alaska (1969).
7. Jasper, J. R. & Evenson, D. F. *Length-Girth, Length-Weight, and Fecundity of Yukon River Chinook Salmon* Oncorhynchus tshawytscha. Alaska Department of Fish & Game, Anchorage, Alaska (2006).
