# Supplementary material for: Recent declines in salmon body size impact ecosystems and fisheries
Source: Nat Commun. 2020 Aug 19;11:4155. doi: 10.1038/s41467-020-17726-z (PMC7438488; doi:10.1038/s41467-020-17726-z)
Supplement: Supplementary file 2 — Reporting Summary [file 41467_2020_17726_MOESM2_ESM.pdf]

natureresearch

Corresponding author(s): Krista Oke, Peter Westley, and Eric Palkovacs

Last updated by author(s): Jul 7, 2020

Reporting Summary

Nature Research wishes to improve the reproducibility of the work that we publish. This form provides structure for consistency and transparency in reporting. For further information on Nature Research policies, see Authors & Referees and the Editorial Policy Checklist .

Statistics

For all statistical analyses, confirm that the following items are present in the figure legend, table legend, main text, or Methods section.

n/a	Confirmed	
☐	☒	The exact sample size (*n*) for each experimental group/condition, given as a discrete number and unit of measurement
☐	☒	A statement on whether measurements were taken from distinct samples or whether the same sample was measured repeatedly
☒	☐	The statistical test(s) used AND whether they are one- or two-sided<br>*Only common tests should be described solely by name; describe more complex techniques in the Methods section.*
☐	☒	A description of all covariates tested
☐	☒	A description of any assumptions or corrections, such as tests of normality and adjustment for multiple comparisons
☐	☒	A full description of the statistical parameters including central tendency (e.g. means) or other basic estimates (e.g. regression coefficient) AND variation (e.g. standard deviation) or associated estimates of uncertainty (e.g. confidence intervals)
☐	☒	For null hypothesis testing, the test statistic (e.g. *F*, *t*, *r*) with confidence intervals, effect sizes, degrees of freedom and *P* value noted<br>*Give P values as exact values whenever suitable.*
☐	☒	For Bayesian analysis, information on the choice of priors and Markov chain Monte Carlo settings
☐	☒	For hierarchical and complex designs, identification of the appropriate level for tests and full reporting of outcomes
☐	☒	Estimates of effect sizes (e.g. Cohen's *d*, Pearson's *r*), indicating how they were calculated

Our web collection on statistics for biologists contains articles on many of the points above.

Software and code

Policy information about availability of computer code

Data collection	No software was used to collect the data in this manuscript, all data were collected by individuals in the field.
Data analysis	R version 3.6.3 was used to analyze the data in this manuscript. Our code is publicly archived and available as a GitHub repository, https://github.com/KristaOke/salmon-size-declines

For manuscripts utilizing custom algorithms or software that are central to the research but not yet described in published literature, software must be made available to editors/reviewers. We strongly encourage code deposition in a community repository (e.g. GitHub). See the Nature Research guidelines for submitting code & software for further information.

Data

Policy information about availability of data

All manuscripts must include a data availability statement. This statement should provide the following information, where applicable:

- Accession codes, unique identifiers, or web links for publicly available datasets
- A list of figures that have associated raw data
- A description of any restrictions on data availability

Our have been publicly archived on the Knowledge Network for Biocomplexity (KNB):
Jeanette Clark, Rich Brenner, and Bert Lewis. 2018. Compiled age, sex, and length data for Alaskan salmon, 1922-2017. Knowledge Network for Biocomplexity. doi:10.5063/F1707ZTM
Krista B Oke, Curry Cunningham, and Peter Westley. 2020. Collated dataset of covariates that could influence body size of Alaska salmon. Knowledge Network for Biocomplexity. doi:10.5063/F1N29V9T

In addition, we used publicly available data from the following sources:
• US Department of Agriculture (USDA), Agricultural Research Service Laboratory. USDA National Nutrient Database for Standard Reference, Legacy Version. Available at: http://www.ars.usda.gov/nutrientdata

- Alaska Department of Fish and Game. Commercial Salmon Fishery Exvessel Prices by Area and Species (2018). Available at: www.adfg.alaska.gov/index.cfm?adfg=commercialbyfisherysalmon.salmoncatch_exvessel (Accessed: 2018-04-23)
- Kibele, J. & Jones, L. Historic air temperatures in Alaska for 1901-2015, with spatial subsetting by region. (2017). doi:10.5063/F1RX997V
- Huang, B. et al. Extended Reconstructed Sea Surface Temperature (ERSST), Version 4. Accessed on April 16, 2018 (2015). doi:10.7289/V5KD1VVF
- Di Lorenzo et al., 2008: North Pacific Gyre Oscillation links ocean climate and ecosystem change, GRL. Available at: http://www.o3d.org/npgo/npgo.php (Accessed: 2018-02-08)
- NOAA, Multivariate ENSO Index. Available at: https://www.esrl.noaa.gov/psd/enso/mei/ (Accessed: 2018-02-08)
- JISAO, Pacific Decadal Oscillation (PDO). Available at: http://research.jisao.washington.edu/pdo/ (Accessed: 2018-02-08)
- NOAA, Bering Sea Ice Cover Index. Available at: beringclimate.noaa.gov (Accessed: 2018-02-08)
- NOAA, Winter Multivariate ENSO Index. Available at: https://www.beringclimate.noaa.gov/data/BCresult.php (Accessed: 2018-02-08)

Field-specific reporting

Please select the one below that is the best fit for your research. If you are not sure, read the appropriate sections before making your selection.

☐ Life sciences ☐ Behavioural & social sciences ☒ Ecological, evolutionary & environmental sciences

For a reference copy of the document with all sections, see nature.com/documents/nr-reporting-summary-flat.pdf

Ecological, evolutionary & environmental sciences study design

All studies must disclose on these points even when the disclosure is negative.

Study description	Six decades of salmon size and age measurements were collected by the Alaska Department of Fish and Game (ADF&G) from 1,014 sampling locations across Alaska. To analyze these data, we used general additive models (GAMs) to quantify spatio-temporal trends in body size for Chinook (Oncorhynchus tshawytscha), chum (O. keta), coho (O. kisutch), and sockeye salmon (O. nerka). We used the chain rule to investigate the extent to which changing age structure (younger age-at-maturity) versus changing growth rates (smaller size-at-age) explain overall variation in salmon size across time. Hierarchical Bayesian models were employed to evaluate evidence in support of three categories of hypothese hypotheses for their ability to explain salmon body size declines—climate change, harvest, and competition with highly abundant wild and hatchery-produced salmon. Finally, we estimated the consequences of changing body size for egg production (population productivity), marine-derived nutrient subsidies to ecosystems, food security for rural and Alaskan Native communities, and commercial fishery harvest value. No new experiments or data collection was performed for this study, with our goal being to synthesize and analyze over 60 years of observations.
Research sample	Data informing these analyses comes from longterm sampling conducted by the Alaska Department of Fish and Game (ADF&G) for management and stock assessment purposes. ADF&G data represent the best available long-term data on salmon body size for the state of Alaska. For this reason the data were an excellent match for our goal of synthesizing salmon body size change. The primary form of these data are length and age measurements collected for over 14 million salmon from four species (sockeye, chum, coho, and Chinook salmon) from across the state of Alaska over 60 years. No new experiments or data collection was performed for this study.
Sampling strategy	Length and age have been sampled by ADF&G for over 60 years. Fish length is collected to the nearest millimeter by ADF&G researchers and technicians using a measuring tape or a manual or electronic measuring board, depending on project and year. Fish age was most commonly estimated from scales based on standardized collection and aging protocols. These aspects of the ADF&G data collection process are detailed in the manuscript. We used all data available from ADF&G and therefore did not conduct power analyses.
Data collection	Data have been collected and recorded in the field by ADF&G staff across the state of Alaska for over 60 years for management purposes.
Timing and spatial scale	Data have been collected for over 60 years across the state of Alaska. Some data gaps occur during this period at some locations due to ADF&G funding and staffing restrictions, but all high quality longterm datasets are included in these analyses.
Data exclusions	Exclusion criteria were determined before statistical analyses were performed. Some data were excluded if: (a) length was measured using a method other than mid-eye to fork of tail, (b) data were duplicated, fell outside of reasonable bounds, or were subject to obvious entry errors, or (c) represented insufficient minimum sample sizes (number of length or age observations) within a given year, or too few years existed for a given location/species combination.
Reproducibility	No experiments were conducted for this study. We took extensive efforts to ensure reproducibly of our research, including archiving all data (on KNB) and all code (a GitHub repository).
Randomization	Not relevant, not experiments were conducted for this study.
Blinding	Not relevant, not experiments were conducted for this study.

Did the study involve field work? ☐ Yes ☒ No

Reporting for specific materials, systems and methods

We require information from authors about some types of materials, experimental systems and methods used in many studies. Here, indicate whether each material, system or method listed is relevant to your study. If you are not sure if a list item applies to your research, read the appropriate section before selecting a response.

Materials & experimental systems

n/a	Involved in the study
☒	☐ Antibodies
☒	☐ Eukaryotic cell lines
☒	☐ Palaeontology
☒	☐ Animals and other organisms
☒	☐ Human research participants
☒	☐ Clinical data

Methods

n/a	Involved in the study
☒	☐ ChIP-seq
☒	☐ Flow cytometry
☒	☐ MRI-based neuroimaging
