# Supplementary material for: Recent declines in salmon body size impact ecosystems and fisheries
Source: Nat Commun. 2020 Aug 19;11:4155. doi: 10.1038/s41467-020-17726-z (PMC7438488; doi:10.1038/s41467-020-17726-z)
Supplement: Supplementary file 3 — Description of Additional Supplementary Files [file 41467_2020_17726_MOESM3_ESM.pdf]

Description of Additional Supplementary Files

File Name: Supplementary Data 1
Description: Estimates of some social consequences for subsistence fisheries users resulting from observed declines in salmon size, by species and population. In particular, we modeled the per-capita change in grams of protein and fat, Calories (kcal), 100g servings, and meals provided. Changes in size compare the average length of a salmon pre-1990 salmon to the average length of a salmon post-2010. Samples sizes are given in Supplementary Data File 4.

File Name: Supplementary Data 2
Description: Estimates of some ecological consequences resulting from observed declines in salmon size, by species and population. In particular, we modeled the per-capita change in grams of phosphorus and female fecundity (number of eggs). Changes in size compare the average length of a salmon pre-1990 salmon to the average length of a salmon post-2010. Samples sizes are given in Supplementary Data File 4.

File Name: Supplementary Data 3
Description: Estimates of an economic consequence for harvesters resulting from observed declines in salmon size, by species and population. In particular, we modeled the per-capita change in price ($USD). Changes in size compare the average length of a salmon pre-1990 salmon to the average length of a salmon post-2010. Samples sizes are given in Supplementary Data File 4, rows with project type "commercial catch"

File Name: Supplementary Data 4
Description: Sample sizes (number of individuals) by species and population for analyses that compare average salmon size pre-1990 to average salmon size post-2010. Number of years included in each period is also provided.

File Name: Supplementary Data 5
Description: Sample sizes (number of individuals) by species and population for general additive models presented in Figure 2. Number of years included is also provided.

File Name: Supplementary Data 6
Description: Sample sizes (number of individuals) by species and population for chain rule analyses presented in Figure 3. Number of years included is also provided.

File Name: Supplementary Data 7
Description: Sample sizes (number of individuals) by species and population for hierarchical Bayesian models presented in Figure 4. Number of years included is also provided.
